# Pectin Synthesis and Pollen Tube Growth in *Arabidopsis* Involves Three GAUT1 Golgi-Anchoring Proteins: GAUT5, GAUT6, and GAUT7

**DOI:** 10.3389/fpls.2020.585774

**Published:** 2020-09-11

**Authors:** Christian Have Lund, Anne Stenbæk, Melani A. Atmodjo, Randi Engelberth Rasmussen, Isabel E. Moller, Simon Matthé Erstad, Ajaya Kumar Biswal, Debra Mohnen, Jozef Mravec, Yumiko Sakuragi

**Affiliations:** ^1^ Department of Plant and Environmental Sciences, Faculty of Science, University of Copenhagen, Frederiksberg, Denmark; ^2^ Department of Biochemistry and Molecular Biology and Complex Carbohydrate Research Center, University of Georgia, Athens, GA, United States

**Keywords:** cell wall, homogalacturonan, galacturonosyltransferase, pollen, male gametophyte, Golgi apparatus****

## Abstract

The major cell wall pectic glycan homogalacturonan (HG) is crucial for plant growth, development, and reproduction. HG synthesis occurs in the Golgi and is catalyzed by members of the galacturonosyltransferase (GAUT) family with GAUT1 being the archetypal and best studied family member. In Arabidopsis suspension culture cells and tobacco leaves, the Golgi localization of Arabidopsis GAUT1 has been shown to require protein-protein interactions with its homolog GAUT7. Here we show that in pollen tubes GAUT5 and GAUT6, homologs of GAUT7, also target GAUT1 to the Golgi apparatus. Pollen tube germination and elongation in double homozygous knock-out mutants (*gaut5 gaut6*, *gaut5 gaut7*, and *gaut6 gaut7*) are moderately impaired, whereas *gaut5* ^−/−^ *gaut6* ^−/−^ *gaut7* ^+/−^ triple mutant is severely impaired and male infertile. Amounts and distributions of methylesterified HG in the pollen tube tip were severely distorted in the double and heterozygous triple mutants. A chimeric protein comprising GAUT1 and a non-cleavable membrane anchor domain was able to partially restore pollen tube germination and elongation and to reverse male sterility in the triple mutant. These results indicate that GAUT5, GAUT6, and GAUT7 are required for synthesis of native HG in growing pollen tubes and have critical roles in pollen tube growth and male fertility in Arabidopsis.

## Introduction

Flowering plants (angiosperms) support a myriad of species on earth and have dominated the terrestrial ecosystem since their diversification over the last 130 million years. One of the biological innovations that occurred in angiosperms was rapid pollen tube germination and elongation, which dramatically shortened the reproductive cycle. Pollen tube growth is restricted to the tip region and involves massive secretion and assembly of newly synthesized cell wall materials, of which HG is a major component (Taylor and Hepler, 1997). The pollen tube apical wall is almost exclusively composed of a pectin network, while the typical load-bearing cellulose-hemicellulose network is only found in the subapical and more distal parts of the tube (Ferguson et al., 1998). HG is a linear homopolymer of α-1,4-linked GalA and is highly esterified during its synthesis in the Golgi apparatus, including methylesterification at the C6 position and, to a lesser extent, acetylesterification at the *O*2 and *O*3 positions of the GalA residue (Perrone et al., 2002; Pauly and Ramírez, 2018). Highly esterified HG is transported in secretory vesicles and deposited at the tip of growing pollen tubes. HG provides both the elasticity necessary for rapid turgor pressure-driven cell expansion and the rigidity required to withstand the turgor (Dardelle et al., 2010; Chebli et al., 2012).

In the Golgi apparatus, the HG backbone is synthesized in the *cis*- and *medial*-cisternae by HG:galacturonosyltransferase (HG : GalAT) and is methyl- and acetyl-esterified by pectin methyltransferases and pectin acetyltransferases (Goubet and Mohnen 1999; Sterling et al., 2001; Caffall and Mohnen, 2009; Atmodjo et al., 2013; Anderson, 2016). *Arabidopsis thaliana* GALACTURONOSYLTRANSFERASE 1 (GAUT1) was the first identified and biochemically characterized HG : GalAT (Sterling et al., 2006). It belongs to the glycosyltransferase family 8 (GT8) of the CAZy Carbohydrate Active Enzyme database (http://www.cazy.org/; Cantarel et al., 2009) and forms the GAUT gene family together with 14 homologs in Arabidopsis (Sterling et al., 2006; Yin et al., 2010a; Yin et al., 2010b). GAUT1 is a protein of 673 amino acids (aa) with canonical type II transmembrane protein topology which includes a short N-terminal cytosolic tail, a single transmembrane anchor domain, a structurally undefined linker region often referred to as the stem region, and a catalytic domain facing the Golgi lumen (Sterling et al., 2006). Biochemical analyses of GAUT1 in Arabidopsis suspension cultured cells revealed that GAUT1 interacts with GAUT7 (AT2G38650), a homolog with 36% aa sequence identity to GAUT1, to form a GAUT1:GAUT7 HG : GalAT complex (Atmodjo et al., 2011). Curiously, the catalytic domain of GAUT1 is cleaved *in vivo* at amino acid position 167 resulting in loss of the single transmembrane domain which generally keeps type II transmembrane proteins in the Golgi. Transient expression studies using fluorescent proteins fused to GAUT1 and GAUT7 in *Nicotiana benthamiana* leaves revealed that GAUT1 remains tethered to the Golgi membrane through protein-protein interactions with GAUT7 (Atmodjo et al., 2011). The Arabidopsis GAUT1:GAUT7 complex in suspension cultured cells was further shown to transiently interact with associating proteins including putative methyltransferases [QUASIMODO3 (QUA3) and AT4G18030], KORRIGAN1, and two homologs of mammalian ribophorins I and II (Atmodjo et al., 2011). These findings shed new light on the biosynthetic components involved in the biosynthesis of HG in suspension cultures and leaves, but raise the question of why nature evolved such a complicated mechanism to target GAUT1 to the Golgi apparatus. Moreover, while recovery of *gaut1* homozygous mutants has not been reported, suggesting a lethal or severely deleterious phenotype, *gaut7* homozygous mutants have no discernable phenotype, leaving open the question of whether alternative protein anchors exist for GAUT1.

Here we report that, in addition to GAUT7, two other homologs of GAUT1, namely GAUT5 (AT2G30575) and GAUT6 (AT1G06780), can also tether GAUT1 in the Golgi apparatus. Our results reveal that the GAUT1-tethering by GAUT5, GAUT6, and GAUT7 is required in pollen for native pollen tube growth, and that lack of this tethering impacts the synthesis and deposition of highly methylesterified HG at the pollen tube apex, which in turn impacts male fertility. The evolution of this GAUT5, GAUT6, GAUT7 tethering mechanism coincided with the emergence of angiosperms.

## Material and Methods

### Plant Materials and Growth Conditions

*Arabidopsis thaliana* ecotype Col-0 was used as WT for all experiments. Seeds of T-DNA insertion lines SALK_ 050186 (*gaut5-1*; located in exon 6), SALK_ 007987 (*gaut6-1*; located in exon 7) and SALK_015189 (*gaut7-1*; located in exon 9) were as described in (Caffall et al., 2009). Plants were grown in soil at a 16 h photoperiod at 100 to 120 µE m^−2^ s^−1^, constant temperature of 20°C, 70% (v/v) relative humidity and watered as necessary. Stable transformation of WT with the promoter-GUS construct, a negative control construct as well as transformation of the *gaut5* ^−/−^ *gaut6* ^−/−^ *gaut7^+^* ^/−^ mutant with the GAUT1 chimera construct and a negative control construct was performed using *Agrobacterium* *tumefaciens* as the vector through the floral dipping method. BASTA^®^ was used to select for positive transformants, except for the promoter-GUS construct for which kanamycin-based selection was used. *Nicotiana benthamiana* was grown in soil at a 16 h photoperiod at 26/24°C (day/night), 60% (v/v) humidity and light intensities of 115 to 150 µE m^−2^ s^−1^. Reciprocal backcrosses of *gaut5* ^−/−^ *gaut6* ^−/−^ *gaut7^+^* ^/−^ plants to WT were carried out by emasculating stage 12 flowers of the female parent plant and subsequently dabbed with anthers from stage 14 flowers (Smyth et al., 1990) of the male parent. Seeds were collected and progeny from each plant were genotyped by PCR for GAUT5, *gaut5*, GAUT7 and *gaut7* using the genotyping primers in **Supplementary Table 2**. Transmission of the *gaut7* allele was analyzed by a χ^2^ test with a significance level of *p* < 0.05. To distinguish between the WT cross-pollination and self-pollination, genotyping was done for WT and mutant allele of GAUT5 because the progeny from the crosses are heterozygous in the *gaut5* allele, while progeny resulting from self-pollination are homozygous in the *gaut5* allele. Three independent attempts to cross the WT (female) with *gaut5* ^−/−^ *gaut6* ^−/−^ *gaut7^+^* ^/−^ (male) failed.

### *In Silico* Analysis

The amino acid sequences of Arabidopsis GAUT1-related family proteins (GAUT1 through GAUT15) were analyzed using the CLC Main Workbench (CLC Bio). Prediction of the transmembrane domain was performing using TMHMM server v. 2.0 (http://www.cbs.dtu.dk/services/TMHMM/).

### Genotyping and RT-PCR Analysis of WT and *gaut* Mutants

Genotyping was done using gene- and T-DNA-specific primer sequences obtained from the T-DNA Primer Design tool provided by the Salk Institute Genomic Analysis Laboratory (http://signal.salk.edu/tdnaprimers.2.html); primer sequences are listed in **Supplementary Table 2**. For RT-PCR analysis, plant materials frozen in liquid nitrogen were ground with a pestle and mRNA extracted using the RNeasy Plant Mini Kit (Qiagen) with on-column DNase treatment according to the manufacturer’s instruction. The integrity of the RNA was confirmed by gel electrophoresis. Before cDNA synthesis, mRNA concentrations were quantified by NanoDrop (ThermoFisher Scientific) and normalized to the equal amount. cDNA was synthesized using the iScript cDNA synthesis kit (Bio-Rad) according to the manufacturer’s protocol. *ACTIN2* (*ACT2*, *AT3G18780*) was used as a reference gene.

### Construction of Fluorescent Protein-Tagged GAUT Proteins and GAUT1 Chimeras

Coding sequences of GAUT1 and GAUT4 to 7 were amplified by gene specific primers (**Supplementary Table 2**) and inserted into the pDONR/zeo vector by the BP reaction following the manufacturer´s instruction (ThermoFisher Scientific). The coding sequences were subsequently transferred to the GATEWAY Expression plasmid pMDC83 by the LR reaction, leading to the generation of GAUTs fused C-terminally with GFP. GAUT1-YFP was generated using the USER cloning strategy as previously described (Sakuragi et al., 2011a; Sakuragi et al., 2011b). The GAUT1 chimeric proteins XYLT : GAUT1 were generated as follows. The different fragments were PCR amplified in the following combinations: GAUT1(168.673) using the primers GAUT1 C-region fwd and GAUT1 C-region rvs; XYLT was amplified using XYLT CTS fwd and XYLT CTS rvs from Col-0 cDNA (see above). Using USER fusion, the fragments were joined and inserted into the pro35S:USER-eGFP vector (Sakuragi et al., 2011) where the chimeras are controlled by 35S promoter and are fused to GFP in frame. The proGAUT1:USER vector was generated by amplifying the promoter region of GAUT1 using the primers proGAUT1 fwd and proGAUT1 USER rvs, in **Supplementary Table 2**, and inserting into pCambia3300u by USER fusion. The proGAUT1 USER rvs primer contains a new USER cloning site for the insertion of the chimeras.

### Promoter-GUS Constructs

The promoter regions cloned were: 1400 bp for *AtGAUT5*, and 1981 bp for *AtGAUT6*, upstream from the start codon. These were used for construction of transgenic lines bearing the *pGAUT5*::*GUS* construct and the *pGAUT6*::*GUS* construct by the procedure as described in (Atmodjo et al., 2011).

### Subcellular Localization of Fluorescently Tagged GAUT Proteins in *N. benthamiana*

The constructs were introduced into *Agrobacterium tumefaciens* and subsequently into *N. benthamiana via Agrobacteria*-mediated heterologous transient expression as previously described (Sakuragi et al., 2011a; Sakuragi et al., 2011b). The viral suppressor p19 was included in all experiments. Confocal laser scanning microscopy was carried out using a Leica SP2 or SP5 microscope using laser excitations at 488 nm, 514 nm, and 587 nm for excitation of GFP, YFP, and mCherry, respectively, and emission detections at 495 to 510 nm, 560 to 600 nm, and 600 to 615 nm, respectively, using 20× magnification water objective. The pinhole diameter was set at 1 airy unit. Scan speed was 800 Hz with a line average of 8. For identification of the subcellular localization, GAUT-GFP fusion proteins were co-expressed with STtmd-YFP, a Golgi marker protein, as previously described (Sakuragi et al., 2011). Co-expression of GAUT1-YFP with GAUT4-GFP, GAUT5-GFP, and GAUT6-GFP was performed as described (Atmodjo et al., 2011). Co-expression of GAUT1-GFP and GAUT7-YFP was performed alongside as the positive control. Single expression of GAUT4-GFP and GAUT7-YFP was also performed alongside as the negative control. For quantification of GFP and YFP signals upon co-expression, areas with characteristic Golgi morphology were manually selected and the pixel mean value within each area in each of the GFP and YFP detection channels was extracted using Leica LCS Lite software (Leica Microsystems). The values were subsequently plotted using OriginPro (OriginLab Corp.) and ANCOVA was performed using MATLAB (The MathWorks, Inc.).

### Yeast-Based Membrane Split-Ubiquitin Assay

The modified split-ubiquitin assay was performed essentially as previously described (Obrdlik et al., 2004; Lund et al., 2015). Briefly, GAUT coding sequences were PCR amplified using primers detailed in **Supplementary Table 2**, and ligated into pBT3-N (bait) and pPR3-N (prey) vectors (Dualsystems Biotech AG, Schlieren, Switzerland) at the *Sfi*I restriction site. Ost1p-NubI, which stably interacts with Cub (Stagljar et al., 1998), was used as the positive control, while the empty NubG vector, pPR3-N, is used as the negative control to detect any unwanted auto-activation of baits. Anp1p, a Golgi-localized yeast enzyme involved in N-glycan biosynthesis (Jungmann and Munro, 1998), was fused to Cub and used as the specificity control to determine the degree of random interaction with NubG-fused proteins (preys). The plasmids were introduced in pairs into yeast strain NMY51 by LiAc transformation. Transformants were selected by SD-Leu-Trp and strains carrying both vectors were grown to an OD_546_ of 1.5. Serial dilutions (between 1- and 1,000-fold) were spotted on SD-Leu-Trp, SD-Leu-Trp-His and SD-Leu-Trp-His-Ade plates. Growth on the SD-His-Leu-Trp and SD-His-Leu-Trp-Ade plates was scored as an indication of interaction.

### Morphological Analysis of Pollen Development and Germination

Pollen viability was assessed using fluorescein diacetate and Alexander staining as previously described (Alexander, 1969; Li, 2011). Stage 12 flowers were fixed in Carnoy’s solution [60% (v/v) ethanol, 30% (v/v) chloroform, 10% (v/v) glacial acetic acid] overnight, dissected and the stamens stained in a drop of Alexander stain solution [11% (v/v) ethanol, 0.01% (w/v) malachite green, 0.05% (w/v) acid fuchsin, 0.005% (w/v) orange G, 4% (v/v) glacial acetic acid, 25% glycerol in ddH_2_O] overnight. Viable pollen grains appear purple, while aborted pollen grains appear green (Alexander, 1969). Pollen germination was examined *in vitro* adapted from the previously-described method (Rodriguez-Enriquez et al., 2013). Briefly, pollen from stage 13 flowers was placed on the surface of a cellophane membrane lying on a microscope slide with an agarose pad [15% (w/v) sucrose, 0.01% H_3_BO_3_ (w/v), 1 mM CaCl_2_·2H_2_O 1 mM Ca(NO_3_)_2_·4H_2_O,1 mM KCl, 1 mM MgCl_2_, and 0.1 mM spermidine, 0.5% (w/v) agarose in ddH_2_O, pH adjusted to 8.0 with KOH]. The slides were incubated in the dark at 24°C for 4 hours at 100% humidity before observation of pollen tube growth using a Leica ICC50 HD microscope (Leica Microsystems, Germany) with 20× magnification. The length of pollen tubes was determined by using ImageJ (https://imagej.nih.gov/ij/).

### Histocytochemistry Analysis

Flowers from 8-week-old transgenic plants were examined for GUS activity after incubating overnight in a GUS staining solution consisting of 50 mM sodium phosphate buffer, pH 7.0, 10 mM ethylenediaminetetraacetic acid, 0.1% (v/v) Triton X-100, 0.5 mM potassium ferricyanide, 0.5 mM potassium ferrocyanide, and 1 mg ml^−1^ 5-bromo-4-chloro-3-indolyl-β-d-glucuronide. Chlorophyll and other pigments were removed with 96% (v/v) ethanol. Multiple T2 plants from at least 5 independent T1 lines showing a consistent staining pattern for each gene were analyzed.

### Immunocytological Analysis of Pollen Tubes and Confocal Microscopy

The cellophane pads with germinated pollen were immediately submerged in 1 ml of 4% formaldehyde in PBS (prepared from paraformaldehyde) in six-well microplates and fixed for 15 min. at room temperature. The pads were washed twice with PBS and blocked with 5% skimmed milk in PBS for 30 min. The blocking solution was exchanged with the primary antibody solution containing JIM7 mAbs (PlantProbes) at 1:10 dilution in blocking solution. After 30 min incubation the pads were washed twice with PBS and incubated with secondary antibody solution consisting of anti-rat AlexaFluor 555-conjugated antibody (Invitrogen) at 1:300 dilution for 30 min. The pads were washed once with PBS and stained with COS^488^ probe (Mravec et al., 2014) at 1:500 dilution in 25 mM MES buffer pH 5.7. After 15 min incubation, the pads were washed with MES buffer and mounted in a drop of Citifluor (Agar Scientific) on a glass slide and covered by a coverslip. The confocal microscopy was performed on Leica SP5 laser-scanning confocal microscope equipped with Argon (488 nm) and HeliumNeon (555 nm) lasers. The pictures were processed with GIMP2 software for overlays and visual enhancement. The signal intensity was measured using ImageJ software.

### Herbicide Susceptibility Test

Arabidopsis seeds were surface sterilized by 10% (v/v) sodium hypochlorite followed by 70% (v/v) ethanol and rinsed five times in sterile water. The sterilized seeds were plated on ½ MS media containing 1% (w/v) sucrose and after vernalization for 2 days in the dark at 4°C were allowed to germinate in a growth chamber for 5 days at 22°C in the diurnal cycle with the photoperiod of 16 h. Seedlings were transferred to ½ MS media containing 1% (w/v) sucrose and 30 µg ml^−1^ phosphinothricine and were incubated for 2 weeks under the same conditions as described above. The numbers of growing and dead seedlings were counted.

### Statistical Analysis

ANOVA (one-way and two-way) and Student´s t-test were used. *p* values < 0.05 is indicated by different lettering or by *.

## Results

### GAUT5 and GAUT6 Are Able to Tether GAUT1 in the Golgi Apparatus Similar to GAUT7

**Figure 1:**
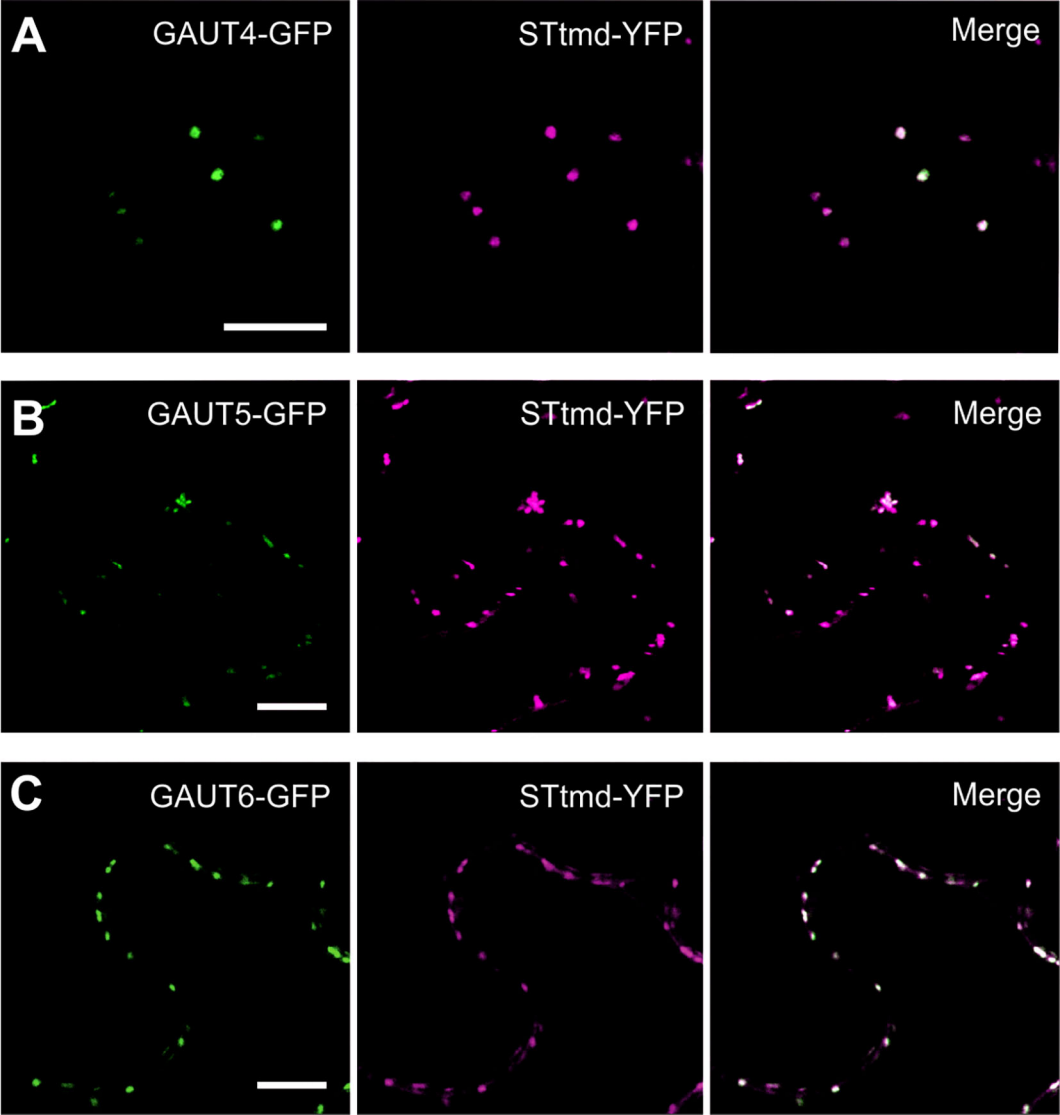
Golgi localization of GAUT4, GAUT5, and GAUT6 GFP fusion cosnstructs. GAUT4-GFP **(A)**, GAUT5-GFP **(B)**, and GAUT6-GFP **(C)** under the control of 35S promoter have been co-expressed with the Golgi marker STtmd-YFP (middle row panels) in *N. benthamiana*. Merge images indicate co-localization (last row panels). Confocal laser scanning microscopy analysis was performed 3 days after transfection. Scale bars = 10 μm.

We sought to test the hypothesis that there are additional GAUT1-anchoring proteins that provide complementary functions to GAUT7. We first screened 11 less characterized Arabidopsis GAUT proteins for their ability to localize to the Golgi apparatus by fusing them to green fluorescent protein (GFP), heterologously expressing them in *Nicotiana benthamiana*, and imaging their expression in the intracellular membrane system by live-cell imaging using confocal laser-scanning microscopy (CLSM) (Sakuragi et al., 2011). We found that GAUT4-GFP, GAUT5-GFP, and GAUT6-GFP showed punctate signals characteristic of the Golgi apparatus (**Figure 1** and **Supplementary Figure 1**, in a manner similar to that previously reported for GAUT7 (Atmodjo et al., 2011). The Golgi localization of these constructs was further confirmed by co-localization with the Golgi marker protein STtmd-YFP (**Figure 1**).

**Figure 2:**
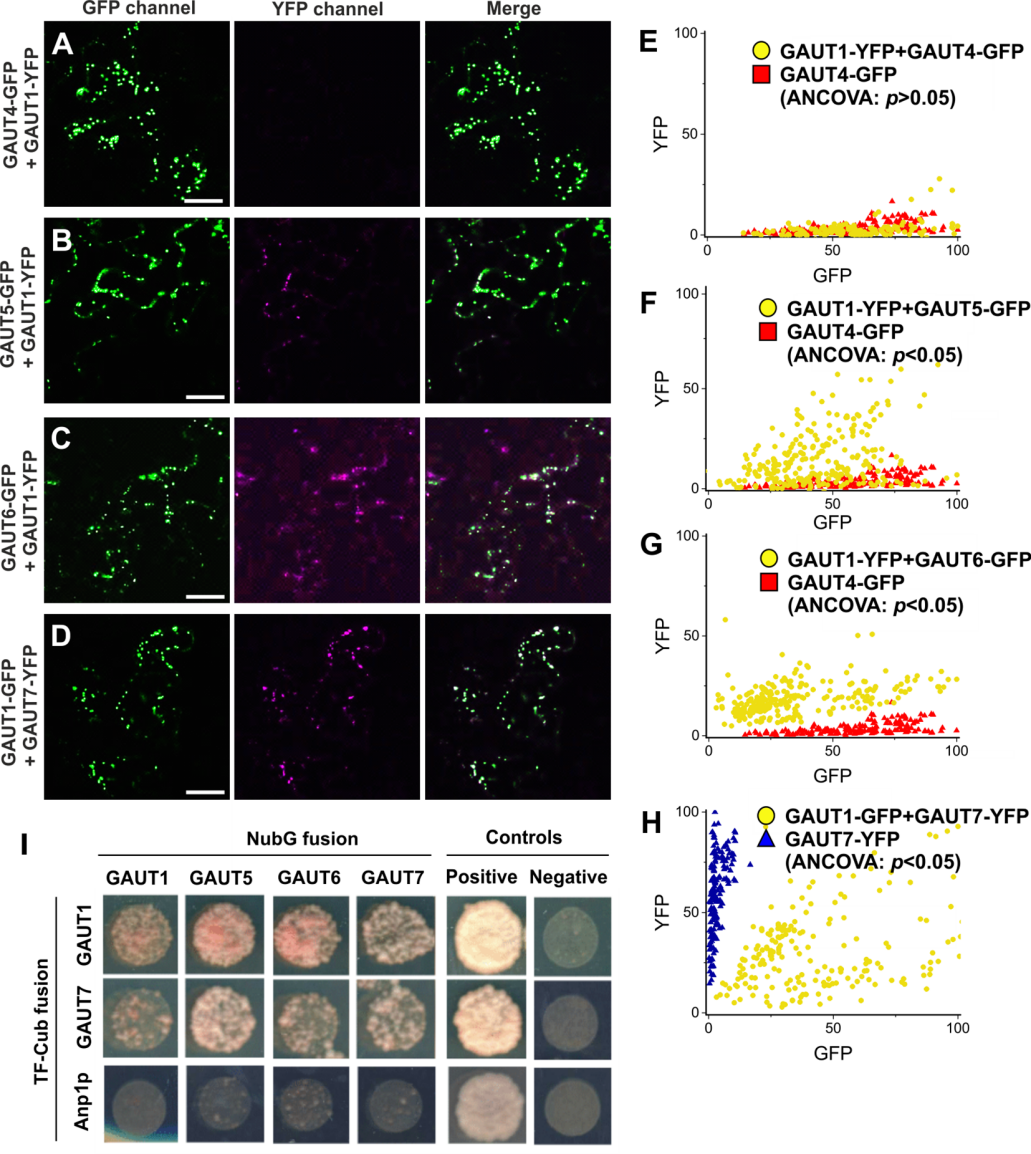
Tethering of GAUT1 by GAUT5, GAUT6, and GAUT7 in the plant Golgi. **(A–D)** Coexpression analysis: GAUT1-YFP co-expressed with **(A)** GAUT4-GFP, **(B)** GAUT5-GFP, **(C)** GAUT6-GFP, and **(D)** GAUT1-GFP co-expressed with GAUT7-YFP as a positive control. Expression of these proteins was performed transiently in *N. benthamiana*. Confocal laser scanning microscopy was performed 3 days after transfection. Scale bars = 10 μm. **(E–H)** Quantifications of GFP and YFP signals (in relative range from 0 to 100, max. signal) in individual punctate signals: GAUT1-YFP co-expressed with **(E)** GAUT4-GFP (n=153), **(F)** GAUT5-GFP (n=153), **(G)** GAUT6-GFP (n = 262), and **(H)** GAUT1-GFP co-expressed with GAUT7-YFP as a positive control (n=195). Confocal laser scanning microscopy was performed 3 days after transfection. *p* values based on ANCOVA are shown. **(I)** Protein-protein interaction assay by yeast-based membrane split-ubiquitin complementation. The yeast growth on the SD-His-Leu-Trp-Ade drop out medium indicates interaction. The positive and negative controls are NubI and pPR3-N, respectively. The Golgi-bound alpha-1,6-mannosyltransferase (Anp1p) was used as specificity control.

We next tested GAUT4, GAUT5, and GAUT6 for their ability to tether GAUT1 to the Golgi. Arabidopsis GAUT1, due to the N-terminal cleavage at aa 167, cannot by itself localize to the Golgi and is consequently secreted out into the apoplast in the absence of an anchoring protein partner such as GAUT7 (Atmodjo et al., 2011). When transiently expressed in *N. benthamiana*, GAUT1-GFP did not by itself yield any detectable GFP fluorescence in the apoplast (likely due to the low pH environment), but did yield punctate Golgi localization signal upon co-expression with GAUT7, as previously reported (Atmodjo et al., 2011). Here we used the same approach, wherein Golgi-retention of GAUT1-YFP by the GAUT candidates was assessed using CLSM upon co-expression of the protein constructs in *N. benthamiana* (**Figure 2** and **Supplementary Figure 1**). Co-expression with either GAUT5-GFP or GAUT6-GFP gave rise to punctate GAUT1-YFP signals that overlapped with the GAUT5-GFP or GAUT6-GFP signals (**Figures 2B, C**). In contrast, co-expression with GAUT4-GFP did not give rise to GAUT1-YFP signals, although punctate GAUT4-GFP signals were clearly visible (**Figure 2A**). Because GFP and YFP have partially overlapping emission wavelengths, it is possible that GFP signals might have crossed over into the YFP channel. To address this, pixel signal intensities in both the YFP and GFP detection channels were obtained for each of the punctate signals and compared to those of the GAUT4-GFP alone, which was used as a control for punctate GFP signals (Atmodjo et al., 2011; **Figures 2E–H**). Signals derived from the GAUT4-GFP control largely distributed along the *x* axis representing GFP signals, indicating limited signal bleed-through between the GFP and YFP detection channels. Signals derived from co-expression of GAUT1-YFP with GAUT4-GFP distributed similarly to the GAUT4-GFP control, which is supported by analysis of co-variance (ANCOVA, *p*>0.05) (**Figure 2E**). In contrast, the signals derived from co-expression of GAUT1-YFP with either GAUT5-GFP or GAUT6-GFP displayed notably different distributions from that of the GAUT4-GFP control (**Figures 2F, G**), more resembling the distribution of GAUT1-YFP and GAUT7-GFP expression (**Figure 1H**). Moreover, the differences between the samples and the GAUT4-GFP control were statistically significantly different (ANCOVA, *p*<0.05). Taken together these results indicate that unlike GAUT4, GAUT5, and GAUT6 were able to influence the GAUT1 trafficking and to retain GAUT1 in the Golgi apparatus *in planta* in a manner similar to that previously reported for GAUT7 (Atmodjo et al., 2011).

To further investigate protein-protein interactions between GAUT1 and GAUT5, GAUT6, and GAUT7, we used a modified yeast-based split-ubiquitin system (Lund et al., 2015) (**Figure 2I**). GAUT1 was N-terminally fused with a reporter consisting of a synthetic transcriptional factor linked to the C-terminal fragment of ubiquitin (TF-Cub) to generate a bait, while GAUT1, GAUT5, and GAUT6, alongside GAUT7 were N-terminally fused with the N-terminal fragment (NubG) of an ubiquitin version bearing an amino acid substitution that prevents irreversible reconstitution of TF-Cub and NubG. Control experiments revealed that TF-Cub-GAUT1 and TF-Cub-GAUT7 proteins were functional because they complemented the yeast growth with the positive control NubI. Further control experiments revealed that NubG-fused GAUT proteins did not promiscuously interact with Anp1p, a yeast Golgi-resident type-II membrane protein, as no complementation of growth was observed. Co-expression of TF-Cub-GAUT1 with NubG-fused GAUTs resulted in complementation in all tested combinations, indicating that GAUT1 interacts with GAUT5, GAUT6, and GAUT7, and also with itself (Atmodjo et al., 2011; Amos et al., 2018). Co-expression of TF-Cub-GAUT7 constructs with NubG-fused GAUTs also resulted in yeast growth in all tested combinations, indicating that GAUT7 can interact with GAUT5 and GAUT6 along with GAUT1 and itself (Atmodjo et al., 2011). The results show that GAUT5 and GAUT6 function similarly to GAUT7 in protein-protein interaction and in retaining GAUT1 in the Golgi apparatus, and thus could compensate for the loss of GAUT7 function in *gaut7* mutant showing no obvious phenotype (Caffall et al., 2009).

### Three GAUT1-anchoring Proteins (GAUT5, GAUT6, GAUT7) Are Required for Male Fertility

The existence of three GAUTs that could serve as anchoring proteins for the HG biosynthetic GAUT1 led us to propose three testable hypotheses for their function. (i) All three GAUTs (GAUT5, GAUT6, GAUT7) are genetically redundant. (ii) Each GAUT has a unique function, and thus, knockout expression of any the GAUTs would lead to a phenotype. (ii) Each GAUT has a unique function *in vivo*, but this function can be partially complemented by the other GAUTs.

**Figure 3:**
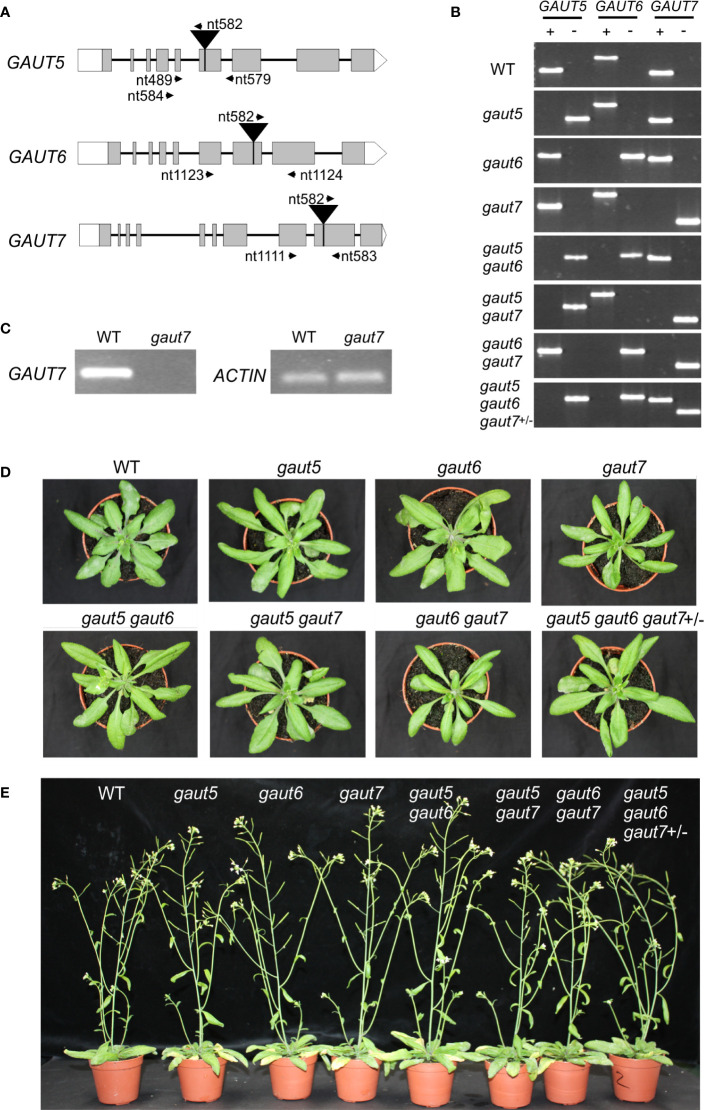
Genotyping and macroscopic phenotypes of the *gaut* single, double, and triple mutants. **(A)** Gene models. T-DNA insertion sites are indicated as black triangle inserts and primer binding sites as black arrows. The sequences of the primers are found in **Supplementary Table 2**. **(B)** PCR-based genotyping. PCR reactions using primer sets targeting the WT loci are indicated as “+,” where those targeting the mutant loci are indicated as “−.” **(C)** *GAUT7* transcript analysis by RT-PCR for WT and *gaut7-1* homozygous plants. Total RNA was isolated from rosette leaves and equal amounts of RNA were used as the template. RT-PCR of the *ACTIN* transcript is shown as a positive control. **(D)** Images of fully expanded rosettes from 8-week-old plants. **(E)** Images of whole 14-week-old plants.

To test these hypotheses, we analyzed the T-DNA insertion mutants of these three genes. Both the *gaut5-1* and *gaut6-1* lines have been previously reported as homozygous knock-out mutants with T-DNA insertions in exons (Caffall et al., 2009). These mutants were confirmed by PCR genotyping and by RT-PCR transcript analysis (**Figures 3A, B** and **Supplementary Figure 3**). The *gaut7-1* homozygous T-DNA insertion line, with an insertion in the 9^th^ exon (**Figures 3A, B**), was previously considered a knock-down mutant because a reduced amount of the *GAUT7* transcript was still detected when a set of oligonucleotide primers that bind upstream of the T-DNA insertion site was used (Caffall and Mohnen, 2009). However, our RT-PCR analysis using a set of primers that bind sequences flanking the T-DNA insertional site did not generate any detectable PCR product in *gaut7-1* (**Figure 3C**). Moreover, *in silico* sequence analysis revealed that the insertion of the T-DNA cassette in the *gaut7-1* line created a stop codon, which would result in a truncated GAUT7 protein consisting of the N-terminal 372 residues out of 674 residues and missing two conserved motifs amongst all GAUT1 family proteins, namely DxD (amino acid positions between 450 and 452) and HxxGxxKPW (amino acid positions between 581 and 589) (**Supplementary Figure 2**). These motifs are expected to coordinate Mn^2+^ and interact with the NDP-sugar donor, respectively (Sterling et al., 2006; Yin et al., 2010a). From these analyses, we concluded that *gaut7-1* is likely a null mutant.

When grown under the standard growth conditions, single mutants *gaut5-1*, *gaut6-1*, and *gaut7-1* (hereafter in this paper referred to as *gaut5, gaut6*, and *gaut7*) grew similarly to WT (**Figures 3D, E**) as previously reported (Caffall et al., 2009). These results showed that each of the three genes is dispensable for the viability of Arabidopsis, which is in contrast to GAUT1 for which no homozygous mutants have yet been reported (Yin et al., 2010b). The lack of a growth phenotype in *gaut5*, *gaut6*, and *gaut7* homozygous mutants supported hypothesis (i), indicating that at least one of the three GAUTs functions redundantly with regards to the overall plant growth.

To further probe whether GAUT5, GAUT6, and GAUT7 had unique physiological roles *in planta*, double mutants of *gaut5*, *gaut6*, and *gaut7* in all combinations were generated by crossing fertile individual single mutants. All the recovered double mutants *gaut5gaut6*, *gaut5gaut7*, and *gaut6gaut7* plants grew normally and were fertile, similar to WT plants (**Figures 3D, E**). This demonstrated that a single functional copy of GAUT5 or GAUT6 or GAUT7 was sufficient for normal plant growth and fertility under greenhouse conditions, further supporting hypothesis (i). For genetic analyses, the heterozygous parental lines *gaut5^+^* ^/−^ *gaut6* ^−/−^ *, gaut5^+^* ^/−^ *gaut7* ^−/−^, and *gaut6* ^−/−^ *gaut7^+^* ^/−^ were obtained and segregation of the respective heterologous alleles was monitored. The Mendelian segregation ratio of 1 (WT): 2 (*gaut5^+^* ^/−^ *gaut6* ^−/−^): 1 (*gaut5* ^−/−^ *gaut6* ^−/−^) and 1 (WT): 2 (*gaut5^+^* ^/−^ *gaut7* ^−/−^): 1 (*gaut5* ^−/−^ *gaut7* ^−/−^) indicated that GAUT7 could fully compensate for the function(s) of GAUT5 and GAUT6 and that GAUT6 could likewise fully compensate for the function of GAUT5 and GAUT7, respectively, in fertilization. Interestingly, however, segregation of *gaut6* ^−/−^ *gaut7^+^* ^/−^ was skewed with a ratio of approximately 4 (WT): 6 (*gaut6* ^−/−^ *gaut7^+^* ^/−^): 1 (*gaut6* ^−/−^ *gaut7* ^−/−^) (**Table 1**), suggesting that GAUT6 and/or GAUT7 had some fertilization-related functions for which GAUT5 could not complement.

**Table 1:** Genetic segregation analysis of *gaut* mutants.

Parents	*n*	Offspring genotypes [observed (expected Mendelian)]		
Self fertilization		WT	*gaut5^+^* ^/−^	*gaut5* ^−/−^
*gaut5^+^* ^/−^ *gaut6* ^−/−^	45	9 (11.25)	26 (22.5)	10 (11.25)
*gaut5^+^* ^/−^ *gaut7* ^−/−^	90	30 (22.5)	39 (45)	21 (22.5)
		WT	*gaut7^+^* ^/−^	*gaut7* ^−/−^
*gaut6* ^−/−^ *gaut7^+^* ^/−^	102	39 (27)	54 (54)	9 (27)*
*gaut5* ^−/−^ *gaut6* ^−/−^ *gaut7^+^* ^/−^	96	50 (24)	46 (48)	0 (24)*
Cross fertilization		WT	*gaut7^+^* ^/−^	
WT (pollen) × *gaut5* ^−/−^ *gaut6* ^−/−^ *gaut7^+^* ^/−^ (ovules)	31	15 (15.5)	16 (15.5)	
T2 population of chimera *pGAUT1*::*XYLT : GAUT1* in *gaut5* ^−/−^ *gaut6* ^−/−^ *gaut7^+^* ^/−^	*n*	Offspring genotypes [observed (expected if rescue)]		
		WT	*gaut7^+^* ^/−^	*gaut7* ^−/−^
line 897-10	68	22 (20.4)	24 (34)	22 (13.6)
line 897-14	65	20 (21.3)	29 (35.5)	22 (14.2)
empty vector control	63	30 (18.6)	32 (31)	0 (12.4) *

Segregation following self-fertilization of double and heterozygous triple mutants and cross-fertilization between WT pollen and gaut5^−/−^ gaut6^−/−^ gaut7^+^^/−^ are shown. Segregation of the gaut7^+^^/−^ alleles in T2 populations of two T1 independent lines bearing GAUT1 chimera construct (designated 897-10 and 897-14) are also shown following self-pollination (see also **Supplementary Figure 8** for the transmission scheme). Progenies were scored by PCR for WT gaut7^+^^/−^ and gaut7^−/−^ and then subjected to a χ^2^ test based on expected Mendelian segregation. p values less than 0.05 are indicated with asterisks. The negative control is gaut5^−/−^ gaut6^−/−^ gaut7^+^^/−^ transformed with the empty vector used to construct the chimera construct.

Efforts to generate the triple mutant failed to yield the triple homozygous *gaut5 gaut6 gaut7* mutant, indicating that the presence of at least one of the three genes is necessary for fertility. A heterozygous *gaut5* ^−/−^ *gaut6* ^−/−^ *gaut7^+^* ^/−^ mutant, however, was able to be isolated and this mutant appeared indistinguishable from WT upon maturity (**Figures 3D, E**). Selfing of this mutant gave a highly-skewed segregation pattern of the *gaut7^+^* ^/−^ alleles in the ratio of 1 (WT): 1 (*gaut7^+^* ^/−^): 0 (*gaut7* ^−/−^) (**Table 1**). This segregation pattern is characteristic of defective transmission of the mutant allele through either the female or male gametophyte (Rédei, 1965).

To ascertain the nature of the gametophyte defect, cross-fertilization was performed between the *gaut5* ^−/−^ *gaut6* ^−/−^ *gaut7^+^* ^/−^ mutant and WT. When the ovules of the heterozygous triple mutant plants were cross-fertilized with pollen from WT plants, the offspring population showed an expected normal segregation ratio 1 (*gaut*7^+^): 1 (*gaut7* ^−^) alleles (**Table 1**), indicating that the female gametophytic transmission in the heterozygous triple mutant is functional. In contrast, when the ovules of WT plants were crossed with pollen from the heterozygous triple mutant, no seed was recovered even after several independent attempts, indicating that male gametophytic transmission through both *gaut5* ^−^ *gaut6* ^−^ *gaut7* ^−^ and *gaut5* ^−^ *gaut6* ^−^ *gaut7^+^* pollen is inhibited during cross pollination, and thus, confirming a role in male fertility. That no seeds were recovered was unexpected, considering that selfed *gaut5* ^−/−^ *gaut6* ^−/−^ *gaut7^+^* ^/−^ mutant could still produce seeds as mentioned above (**Table 1**). We speculate that, unlike during native selfing when transmission of paternal *gaut5* ^−^ *gaut6* ^−^ *gaut7^+^* allelic combination is functional, during crosspollination external stress and dosage dependent effects of the heterozygous *gaut7^+^* ^/−^ genotype in the paternal tissues and/or premeiotic microsporocytes affect pollination. Monosaccharide compositions of cell walls isolated from rosette leaves from wild type and the different mutants revealed no differences in most sugars between WT and all the homozygous single, homozygous double, and *gaut5* ^−/−^ *gaut6* ^−/−^ *gaut7^+^* ^/−^ triple mutant (**Supplementary Figure 4**). The only exceptions were a slightly elevated GalA content in the *gaut5* single mutant, a concomitant reduction in the Glu and Gal content, and a slight elevation in the Xyl+Man content in *gaut7*, *gaut5* *gaut6*, *gaut5 gaut7*, *gaut6 gaut7*, and *gaut5* ^−/−^ *gaut6* ^−/−^ *gaut7^+^* ^/−^ mutants. Taken together the combined results suggested that although they might have redundant functions in vegetative tissue, the three GAUTs either had unique functions or could partially complement for each other during fertilization in the reproductive tissues, thus supporting hypothesis (ii) or (iii).

### GAUT5, GAUT6, and GAUT7 Are Expressed in Pollen

**Figure 4:**
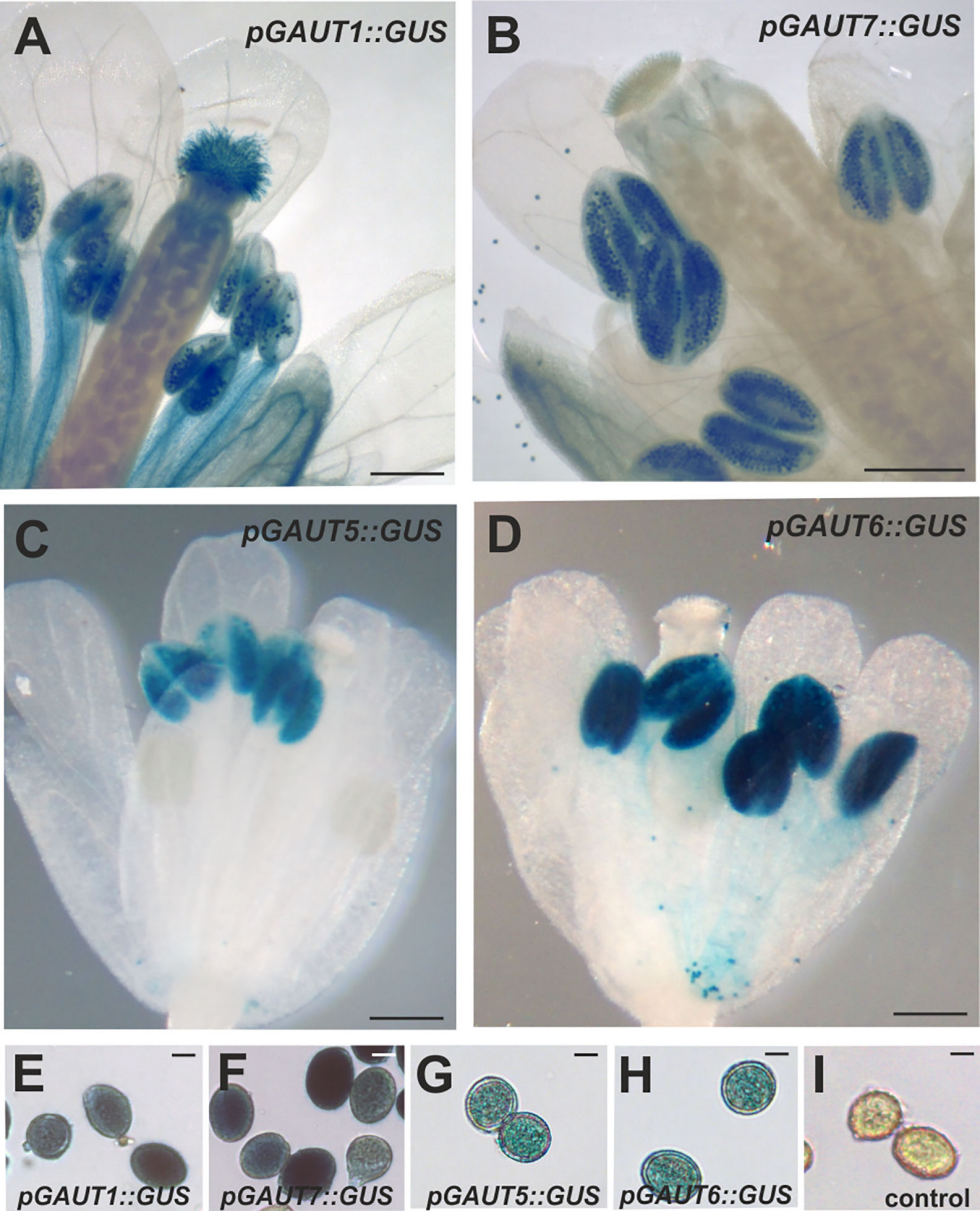
Activity of *GAUT1*, *GAUT5*, *GAUT6*, and *GAUT7* promoters in flowers. WT plants were transformed with the genomic regions upstream of the *GAUT5* gene and the *GAUT6* gene fused with the β-glucuronidase (*GUS*) gene and T2 plants were analyzed as described in *Materials and Methods*. **(A–D)** GUS staining of the whole flowers, of *pGAUT1*::*GUS* **(A)**, *pGAUT7*::*GUS* (Atmodjo et al., 2011) **(B)**, *pGAUT5*::*GUS* **(C)** and *pGAUT6*::*GUS* **(D)**. Scale bars = 1 mm. **(E–I)** GUS activity in pollen of *pGAUT1*::*GUS* **(E)**, *pGAUT7*::*GUS* **(F)**, *pGAUT5*::*GUS* **(G)** and *pGAUT6*::*GUS* line **(H)**. **(I)** Pollen from a negative control plant bearing the empty *GUS* vector. Scale bars = 10 μm.

Consistent with the defects in the male transmission, the *GAUT6* and *GAUT7* transcripts have been detected in mature pollen as well as in pollen tubes by Arabidopsis ATH1 microarray analysis (Wang et al., 2008; Qin et al., 2009). While the *GAUT5* transcript was not analyzed in the microarray due to the lack of probes against this transcript, an mRNA sequencing study of mature pollen revealed that *GAUT5* transcript is also present (Loraine et al., 2013). Moreover, proteomic analyses of pollen have detected the presence of GAUT1, GAUT5, GAUT6, and GAUT7 proteins (Grobei et al., 2009; Pep2Pro, http://fgcz-pep2pro.uzh.ch/). We also analyzed the promoter activity of the four *GAUT* genes using promoter-*GUS* constructs (**Figure 4**). GUS staining revealed partially overlapping patterns in flowers. While the promoter activity of *GAUT1* was prominent in several tissues including petals, anthers, anther filaments, stigma and pollen, those of *GAUT5, GAUT6*, and *GAUT7* were prominently seen in anther and specifically in the pollen grains (**Figures 4A–H**). The transcript and protein expression profiles are therefore consistent with a role of these GAUTs in pollen formation and/or function.

### GAUT5, GAUT6, and GAUT7 Are Required for Pollen Germination and Elongation

The results from genetic analyses led us to investigate the pollen. We first tested the viability of pollen in the anthers. Staining with fluorescein diacetate (Li, 2011) showed the same level of staining in pollen grains from WT and in pollen from heterozygous triple *gaut5 gaut6 gaut7^+^* ^/−^ mutant plants (**Supplementary Figure 5A**). Alexander staining (Alexander, 1969; Robertson et al., 2004) also yielded a purple colorization of both WT and *gaut5* ^−/−^ *gaut6* ^−/−^ *gaut7^+^* ^/−^ mutant-derived pollen (**Supplementary Figure 5B**). These results confirmed that the pollen heterozygous triple mutant is comparably viable as that of WT.

**Figure 5:**
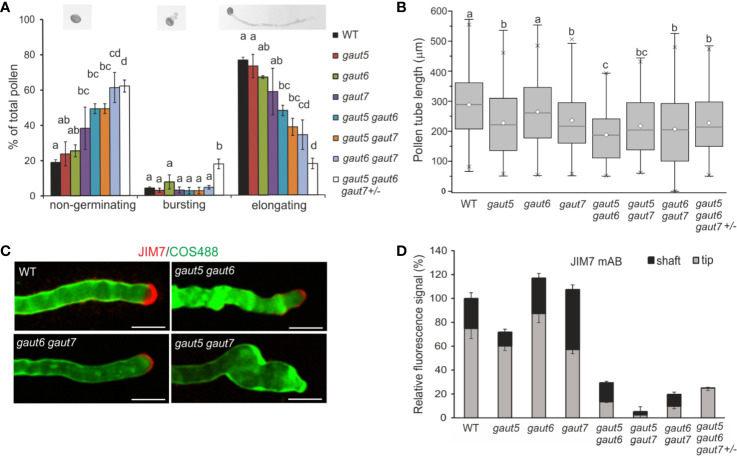
Pollen tube germination and elongation of the *gaut5*, *gaut6*, *gaut7* homozygous single, homozygous double, and triple mutants (*gaut5* ^−/−^ *gaut6* ^−/−^ *gaut7^+^* ^/−^). **(A)** Distributions of pollen germination phenotypes: “non-germinating” pollen, “bursting” pollen, and pollen with “elongating” tubes (n>100). Different letters indicate significant differences as determined by One-way ANOVA (*p*<0.05). **(B)** The pollen tube lengths were measured *in vitro* following 4 hours of pollen germination (n>100). **(C)** Double labeling of lowly methylesterified HG using the COS^488^ probe (green) and of highly methylesterified HG with JIM7 mAb (red). Instances of complete lack of JIM7 was visible for *gaut5 gaut7* double mutant. Scale bars = 10 µm. **(D)** Quantification of the signals after labeling HG in the pollen tip and shaft regions with JIM7 (n ≥ 5, SEM), expressed as relative to the level in the WT pollen tip.

We next studied pollen germination and pollen tube growth *in vitro* as previously described (Rodriguez-Enriquez et al., 2013). Pollen from newly opening flowers of all genotypes were grown simultaneously in a temperature and humidity-controlled growth chamber and analyzed. The WT and *gaut5* ^−/−^ *gaut6* ^−/−^ *gaut7^+^* ^/−^ mutant displayed significant differences in *in vitro* pollen germination (**Figure 5A**). A large proportion (~60%) of the heterozygous triple mutant pollen did not germinate and nearly half of those that did germinate displayed bursting tubes. On the contrary, almost 80% of WT pollen successfully germinated and only 5% of these showed burst pollen tubes. Some of the single and double homozygous mutants displayed intermediate levels of pollen germination. While the *gaut5* and *gaut6* single mutants displayed minor, non-significant increases in the proportion of non-germinating pollen, the *gaut7* single mutant displayed a significant increase in the number of non-germinating pollen grains. Furthermore, all the double mutants displayed additive effects of their respective single mutants (**Figure 5A**). These results indicate that GAUT7 is required for WT-like germination and that GAUT6 and GAUT5 also function in pollen germination. In contrast to the *gaut5* ^−/−^ *gaut6* ^−/−^ *gaut7^+^* ^/−^ triple mutant, the proportion of bursting pollen tubes was not altered in the single and double homozygous mutants compared to WT, supporting some level of redundancy in GAUT5, GAUT6, and GAUT7 function in maintaining pollen tube strength.

Measurement of pollen tube length after 4 hours of germination revealed marked differences across the genotypes (**Figure 5B**). The mean length of the WT pollen was 290 μm. While the mean length of the *gaut6* mutant pollen tube was indistinguishable from that of WT, those of *gaut5* and *gaut7* mutants were significantly shorter (220 μm and 210 μm, respectively; *p*<0.05), suggesting a role of GAUT5 and GAUT7 in pollen tube elongation. All the double mutants and the heterozygous triple mutants also had significantly shorter pollen tubes (~210 μm) than WT (*p*<0.05). Taken all together, these results demonstrated that the absence of GAUT5, GAUT6, and GAUT7 significantly impaired the germination of pollen and subsequent growth of the pollen tube. This was especially severe in the *gaut5* ^−/−^ *gaut6* ^−/−^ *gaut7^+^* ^/−^ mutant that consequently resulted in male infertility. GAUT5, GAUT6, and GAUT7 operate in the deposition of HG in the pollen tube cell wall.

In order to investigate the roles of GAUT5, GAUT6, and GAUT7 in HG synthesis in pollen tubes, we performed immuno-localization using a monoclonal antibody (mAb) JIM7 that recognizes HG with a high degree of esterification—the form of HG synthesized in the Golgi and secreted at the tip of the pollen tube (Chebli et al., 2012; **Figure 5C** and **Supplementary Figure 6**). Quantification of the mAb-generated signals from the tip and the shaft region of the growing pollen tube (**Figure 5D**) showed that in the case of the *gaut5, gaut6*, and *gaut7* single mutants, the signal was reduced compared to WT, although to a different extent among the different *gaut* mutants. In the case of *gaut7*, the signal was visibly mislocalized with more equally distributed signal along the whole pollen tube (**Supplementary Figure 6**). In comparison, the *gaut5* mutant retained JIM7 signal primarily at the tip with much reduced signal on the shaft, while the *gaut6* mutant had overall similar, but slightly reduced, tip to shaft staining as the WT. These results suggest that the different GAUTs may affect the amount and location of methylesterified HG in the growing pollen tube. JIM7 binding at the tip and the shaft was almost completely abolished in all double and *gaut5* ^−/−^ *gaut6* ^−/−^ *gaut7^+^* ^/−^ triple mutant (**Supplementary Figure 6**), demonstrating the critical importance of GAUT5, GAUT6, and GAUT7 in anchoring GAUT1 for synthesis of methyl-esterified HG in growing pollen tubes. Altered fluorescent intensity and distribution were also observed in the double mutants labeled with COS^488^ (chitosan oligosaccharides aminooxy-functionalized with Alexa Fluor fluorophore; Mravec et al., 2014), which is a probe specific for HG with a low degree of esterification (similar to the commonly used JIM5 mAb) and labels the subapical and shaft region of WT pollen tubes (**Figure 5C**). Taken together, the results indicate that the GAUT1-tethering proteins are required for normal synthesis and deposition of methylesterified HG at the pollen apex and that their loss affects the distribution of methylesterified HG along the pollen tube shaft in GAUT homolog-specific manners. These results support hypothesis III and indicate that each GAUT has a unique function that is partially complemented by the other GAUTs.

### A Chimeric, Golgi-Localized GAUT1 Restores Male Fertility in the Heterozygous Triple *gaut* Mutant

**Figure 6:**
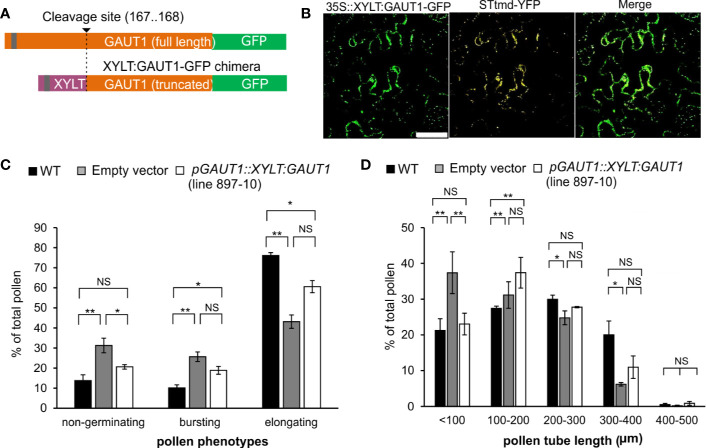
The *pGAUT1::XYLT : GAUT1* chimera restores pollen tube elongation in the *gaut5 gaut6 gaut7^+^* ^/−^ mutant. **(A)** Design of the GAUT1 chimera construct. The assumed proteolytic cleavage site is indicated. **(B)** Golgi-localization of the *35S:XYLT : GAUT1* chimera. The construct was expressed transiently in *N. benthamiana* together with the Golgi marker STtmd-YFP. Scale bar = 50 µm. **(C)** Analysis of the rescue of the pollen phenotypes by *pGAUT1::XYLT : GAUT1* chimera construct in *gaut5* ^−/−^ *gaut6* ^−/−^ *gaut7* ^+/−^ mutant (line 897-10). The empty vector in the same mutant was used as a negative control. Distributions of pollen germination phenotypes: “non-germinating” pollen, “bursting” pollen, and pollen with “elongating” tubes (more than 330 pollen of three plants were counted; SEM). **(D)** Quantification of the length of the pollen tubes (n≥ 120; SEM). * and ** indicate significant differences as determined by One-way ANOVA (*p*<0.05 and *p*<0.01, respectively). NS indicates not significantly different.

Based on the previous results we hypothesized that the severe pollen-related phenotypes were caused by mislocalization of GAUT1 due to the absence of the three anchoring GAUTs (GAUT5, GAUT6, GAUT7), resulting in altered HG synthesis. To test this, we attempted to trace the localization of GAUT1 C-terminally fused with mCherry in the series of the *gaut* mutants. In *N. benthamiana*, GAUT1-mCherry localized to the cell periphery (**Supplementary Figure 7**). These results suggest that the endogenous leaf-expressed GAUTs of tobacco are not able to tether the introduced Arabidopsis GAUT1-mCherry to the Golgi apparatus. Furthermore, the localization of GAUT1-mCherry in Arabidopsis under the control of the native GAUT1 promoter failed due to insufficient signals above the noise, most likely because of low levels of native GAUT1expression. We thus took an alternative approach by creating a GAUT1 construct capable of accumulating in the Golgi apparatus independently of GAUT5, GAUT6, and GAUT7 and asked whether expression of such a construct would enable recovery of homozygous triple mutant *gaut5 gaut6 gaut7* plants. To accomplish this, the C-terminal region of GAUT1 (amino acid positions between 168 and 673) containing the presumed catalytic domain was fused with the N-terminal sequence (amino acid positions between 1 and 90) of a β-1,2-xylosyltransferase (XYLT, At5g55500), a glycosyltransferase involved in *N*-glycosylation (**Figure 6A**). This N-terminal sequence was previously shown to be sufficient for localization in the medial Golgi cisternae (Schoberer et al., 2013). The subcellular localization of the chimeric GAUT1 was analyzed by heterologous expression in *N. benthamiana* using a 35S promoter to drive its expression (**Figure 6B**). Punctate fluorescence signals were observed for the chimeric GAUT1 construct which co-localized with the Golgi marker STtmd-YFP (Munro, 1995; Saint-Jore-Dupas et al., 2004; Sakuragi et al., 2011). These results show that the chimeric GAUT1 is able to localize to the Golgi apparatus.

To test whether the chimeric GAUT1 protein was able to rescue the phenotype of multiple *gaut* mutant pollen, we created a construct where the chimeric GAUT1 was expressed from the native GAUT1 promoter. These constructs were introduced into the Arabidopsis *gaut5* ^−/−^ *gaut6* ^−/−^ *gaut7^+^* ^/−^ mutant *via* stable transformation and the segregation pattern of the *GAUT7* and *gaut7* alleles was analyzed by PCR in T2 generation. If the chimeric GAUT1 restores the male fertility, the segregation pattern of 3 (WT): 5 (*gaut7^+^* ^/−^): 2 (*gaut7* ^−/−^) was expected (**Supplementary Figure 8**). The T2 population derived from the control line, wherein the empty vector was inserted, maintained the same transmission frequencies as observed for the untransformed *gaut5* ^−/−^ *gaut6* ^−/−^ *gaut7^+^* ^/−^ triple mutant (**Table 1**). On the other hand, the T2 populations from two independent lines bearing the chimeric construct (lines 897-10 and 897-14) segregated in skewed ratios (22:24:22 and 20:29:22) (**Table 1**) and, importantly, we were able to recover rescued homozygous triple *gaut5 gaut6 gaut7* mutants. These results confirm that the previous inability to recover homozygous *gaut5 gaut6 gaut7* triple mutants is due to a lack of GAUT1-synthesized HG due to a lack of retention of GAUT1 in the Golgi.

We further analyzed the control, and the chimera-containing 897-10 T1 line for *in vitro* pollen germination and pollen tube elongation. In this line the high proportion of defective pollen (“non-germinating”) observed in the negative control line (**Figure 6C**) was complemented to WT-levels. The low proportion of elongating pollen and the increased proportion of bursting pollen, however, remained similar to the negative control level. Partial complementation was also observed for the length of elongating pollen tubes (**Figure 6D**). These results suggest that the GAUT1 chimera, at least partially, is able to complement the loss of function of the three GAUT anchor proteins.

## Discussion

Homogalacturonan, the most abundant and structurally simplest pectin, has roles in many stages of plant vegetative and reproductive development. While the current understanding of how HG is synthesized *in planta* by the different members of the GAUT gene family is far from complete, it is becoming increasingly clear that the different members of the gene family produce HG with unique functions (Atmodjo et al., 2013; Amos et al., 2018; Biswal et al., 2018). Here we showed that three different GAUT proteins, GAUT5, GAUT6, and GAUT7, interact with GAUT1, and thereby tether and retain the N-terminally cleaved HG : GalAT to the Golgi apparatus, the location of HG synthesis. This function of the three GAUTs appears to be redundant during the vegetative growth stage, as demonstrated by the WT-like growth of the homozygous single, homozygous double, and even the heterozygous triple mutant plants. However, they are critically required for normal reproduction, specifically in the male gametophyte. The *gaut5* ^−/−^ *gaut6* ^−/−^ *gaut7^+^* ^/−^ triple mutant lacking these GAUT1-anchoring proteins was severely defective in pollen germination and tube elongation. A GAUT1 chimera protein capable of self-tethering to the Golgi apparatus was able to partially restore the pollen functions. These observations show that GAUT5, GAUT6, and GAUT7 each tether GAUT1 in the Golgi enabling HG synthesis in the pollen tube.

Mutation of GAUT5, GAUT6, and GAUT7 have differing effects on the deposition of methylesterified HG at the apex of pollen tubes, a process critical for native pollen tube growth and male fertility. The binding pattern of the mAb JIM 7, which recognizes highly methylesterified HG, was altered in the growing pollen tubes of all the mutants, even the homozygous *gaut5*, *gaut6*, and *gaut7* single mutants. The defect was increasingly more severe in the homozygous double and heterozygous triple mutants, resulting in significantly reduced pollen germination and pollen tube growth, and in infertility in the *gaut5* ^−/−^ *gaut6* ^−/−^ *gaut7^+^* ^/−^ mutant leading to the inability to isolate *gaut5* ^−/−^ *gaut6* ^−/−^ *gaut7* ^−/−^ homozygous triple mutant plants. The introduction of a GAUT1 chimera protein capable of self-tethering to the Golgi apparatus allowed a successful recovery of the rescued homozygous triple mutant, thus confirming the importance of GAUT5, GAUT6, and GAUT7 function to localize GAUT1 to the Golgi for HG biosynthesis and male gametophyte development.

Growing pollen tubes, as single cells with fast tip-growth facilitated mainly by HG, provide a unique system to start teasing apart the biological functions imparted by GAUT1, GAUT5, GAUT6, GAUT7, their protein:protein interactions and the resulting protein complexes. This task would be much more difficult to carry out using other plant parts that consist of multiple different tissue and cell types. Almost all the Arabidopsis GAUTs are expressed at some level in pollen (Loraine et al., 2013), the most highly expressed being GAUT13 and GAUT14 whose functions in HG synthesis in pollen and pollen tube growth have previously been demonstrated (Wang et al., 2013). It is interesting to note that in pollen, when compared to the transcript levels of GAUT13 and 14, GAUT1 transcript level was reported to be 6 to 9 times lower, and those of GAUT5, GAUT6, and GAUT7 were even lower than GAUT1 (Loraine et al., 2013), yet these three proteins still play significant roles in ensuring a proper plant reproduction process. It would be interesting to investigate the precise roles of GAUT13 and GAUT14 versus the GAUT1-containing HG : GalAT complexes in HG biosynthesis in pollen and pollen tubes.

We envisage that the diversified GAUT protein tethering mechanism can expand the biosynthetic capabilities of GAUT1. It was previously shown that GAUT1 and GAUT7 form the core of an HG : GalAT complex, which transiently interacts with a number of associating proteins including putative methyltransferases in Arabidopsis suspension culture cells (Atmodjo et al., 2011). Furthermore, a recent detailed biochemical analyses of heterologously expressed GAUT1:GAUT7 complex revealed that GAUT1 alone expresses rather poorly compared to when it is co-expressed with GAUT7, and that the HG : GalAT activity of GAUT1 alone is much lower compared to the GAUT1:GAUT7 complex (Amos et al., 2018). Based on these previous findings, we proposed two possibilities for how GAUT1-anchoring by GAUT5, GAUT6, and GAUT7 may have significance beyond Golgi localization. These categories may not be mutually exclusive, but may overlap with each other.

One possibility is that the interaction and/or complex formation with GAUT5, GAUT6, and GAUT7 may impart changes in GAUT1 enzymatic function, with regards to, for example, enzyme kinetics and/or HG product size. Yin et al. (2010b) pointed out that GAUT5 and GAUT7, but not GAUT6, lack a potential nucleophilic threonine residue in the predicted catalytic site (**Supplementary Figure 2**). It is therefore possible that GAUT5 and GAUT7 play a non-catalytic structural role, while GAUT6 may have a transferase activity. The latter may function as a catalytic subunit in the synthesis of HG backbone, either in association with GAUT1 or independently. Thus, multiple forms of catalytic cores may exist, consisting of GAUT1 alone, GAUT1:GAUT6 complex, or GAUT6 alone, and each of these catalytic core could further associate with a structural subunit (e.g., GAUT5 and GAUT7). Furthermore, the tethering proteins could control the physical location of GAUT1 within the Golgi cisternae. For instance, these proteins appear to share a common motif, Arg-Arg-Trp-x-ArgR/Lys, in the N-terminal domain localized to the cytosol (**Supplementary Figure 2**). Varieties of arginine-based motifs have been shown to affect the dynamics of the trafficking of type II membrane proteins between ER and Golgi and possibly within the Golgi cisternae (Dancourt and Barlowe, 2010; Banfield, 2011). It is interesting to note that GAUT7 possesses 12 consecutive Gly residues adjacent to the motif. The function of the Gly residue stretch remains to be elucidated, but it might influence the trafficking of GAUT7 in and around the Golgi apparatus differently from GAUT5 and GAUT6, and thus, affect the precise structure of the synthesized HG. Determination of the precise unique molecular functions of GAUT5, GAUT6, and GAUT7 will require *in vitro* reconstitution and enzyme kinetics studies of the different GAUT1-containing complexes, as well as analysis of HG produced by the different complexes.

The second possibility is that the different HG biosynthetic GAUT1-containing complexes may interact with distinct sets of associating proteins in the Golgi, during HG trafficking through the secretory pathway, and/or *in muro*. For example, GAUT5, GAUT6, and GAUT7 may each mediate interaction of the GAUT1 HG : GalAT complexes with different pectin methyltransferases (Atmodjo et al., 2011) and/or pectin acetyltransferases in the Golgi apparatus, resulting in HG products with potentially diverse degrees and/or patterns of esterification. GAUT5, GAUT6, and GAUT7 might also influence interaction of GAUTs with different vesicle trafficking proteins, resulting in transport of different HG products to specific destinations at the cell periphery for deposition into the wall. Such possibilities are in agreement with how the single and double *gaut* mutants affect the degree and pattern of mAb JIM7 binding in the pollen tip and shaft. Upon incorporation into the wall, the newly deposited, highly methylesterified HG may be recognized as a preferred substrate, based on the specific degree and pattern of esterification, by specific pectin methyl esterases and other pectin modifying enzymes. Indeed, transcriptomics and proteomics studies have shown that at least 14 pectin methylesterases (PMEs), 21 pectin methylesterase inhibitors (PMEIs), and 16 pectin lyases/pectin lyase-like proteins (PLLs) are highly and/or specifically expressed in pollen and pollen tubes (Loraine et al., 2013; Mollet et al., 2013), indicating the importance of HG modification for normal male gametophytic transmission. Determination of the unique molecular functions of GAUT5, GAUT6, and GAUT7 will require identification of the associating protein partners of the different GAUT1-containing protein complexes and the HG substrate specificities of the diverse pectin modifying enzymes.

Rapid pollen tube germination and elongation is a hallmark of angiosperms, which dramatically shortened the reproductive cycle and enabled angiosperms to evolve and diversify. As HG plays a central role in pollen tube germination and elongation, the evolution of a multi GAUT-mediated HG synthesis strategy might have provided a competitive advantage for the rate and control of HG synthesis in the pollen tube, thereby supporting rapid pollen tube growth and structural integrity during it race through the pistil to the egg.

In conclusion, we discovered the existence of previously unappreciated complexity in the biosynthesis of one of the most structurally simple cell wall polysaccharides in pollen tubes. The results underscore the complexity and need for rapid biosynthesis and deposition of HG in the highly dynamic male gametophyte in flowering plants. This work paves the way for future studies aimed at deciphering the molecular mechanisms of how the different GAUT anchors and catalytic subunits achieve rapid HG synthesis and support rapid pollen tube growth.

## Data Availability Statement

All datasets presented in this study are included in the article/supplementary material.

## Author Contributions

YS, CL, AS, and JM conceived the experiment, and together with MA, AB, SE, DM, IM, and RR carried it out. YS, CL, AS, JM, MA, AB, and RR designed and carried out the data analysis. YS, CL, MA, AB, DM, and JM wrote and edited the manuscript. All authors contributed to the article and approved the submitted version.

## Funding

We thank the Danish Council for Independent Research (project no. 272-07-0152 and 8022-00222B), the Villum Fonden (projects no. 13363 and 17489), and the Center for Bioenergy Innovation, a U.S. Department of Energy Bioenergy Research Center supported by the Office of Biological and Environmental Research in the DOE Office of Science, for funding.

## Conflict of Interest

The authors declare that the research was conducted in the absence of any commercial or financial relationships that could be construed as a potential conflict of interest.
