# Supplementary material for: Pectin Synthesis and Pollen Tube Growth in Arabidopsis Involves Three GAUT1 Golgi-Anchoring Proteins: GAUT5, GAUT6, and GAUT7
Source: Front Plant Sci. 2020 Sep 11;11:585774. doi: 10.3389/fpls.2020.585774 (PMC7533613; doi:10.3389/fpls.2020.585774)
Supplement: Supplementary file 1 [file DataSheet_1.pdf]

Supplementary Figure 1

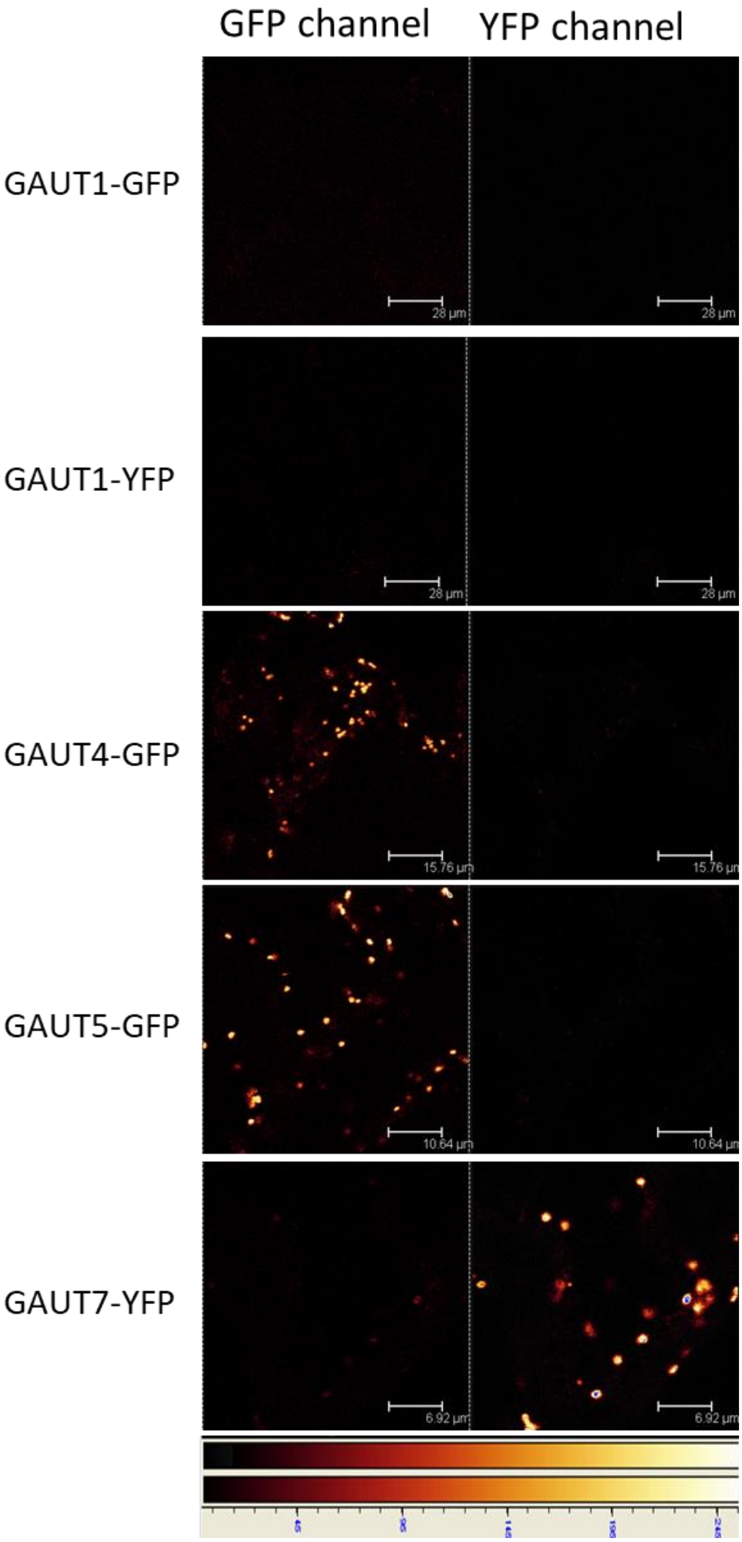

Supplementary Figure 1. Localization analysis of GAUT GFP/YFP fusion proteins and test for signal cross-bleeding of GFP and YFP channels.
GAUT1-GFP, GAUT1-YFP, GAUT4-GFP, GAUT5-GFP, GAUT7-GFP constructs under the control of 35S promoter were transiently expressed in N. benthamiana. CSLM analysis was performed 3 days after transfection. Recorded emission within the GFP and YFP channels is shown.

Supplementary Figure 2

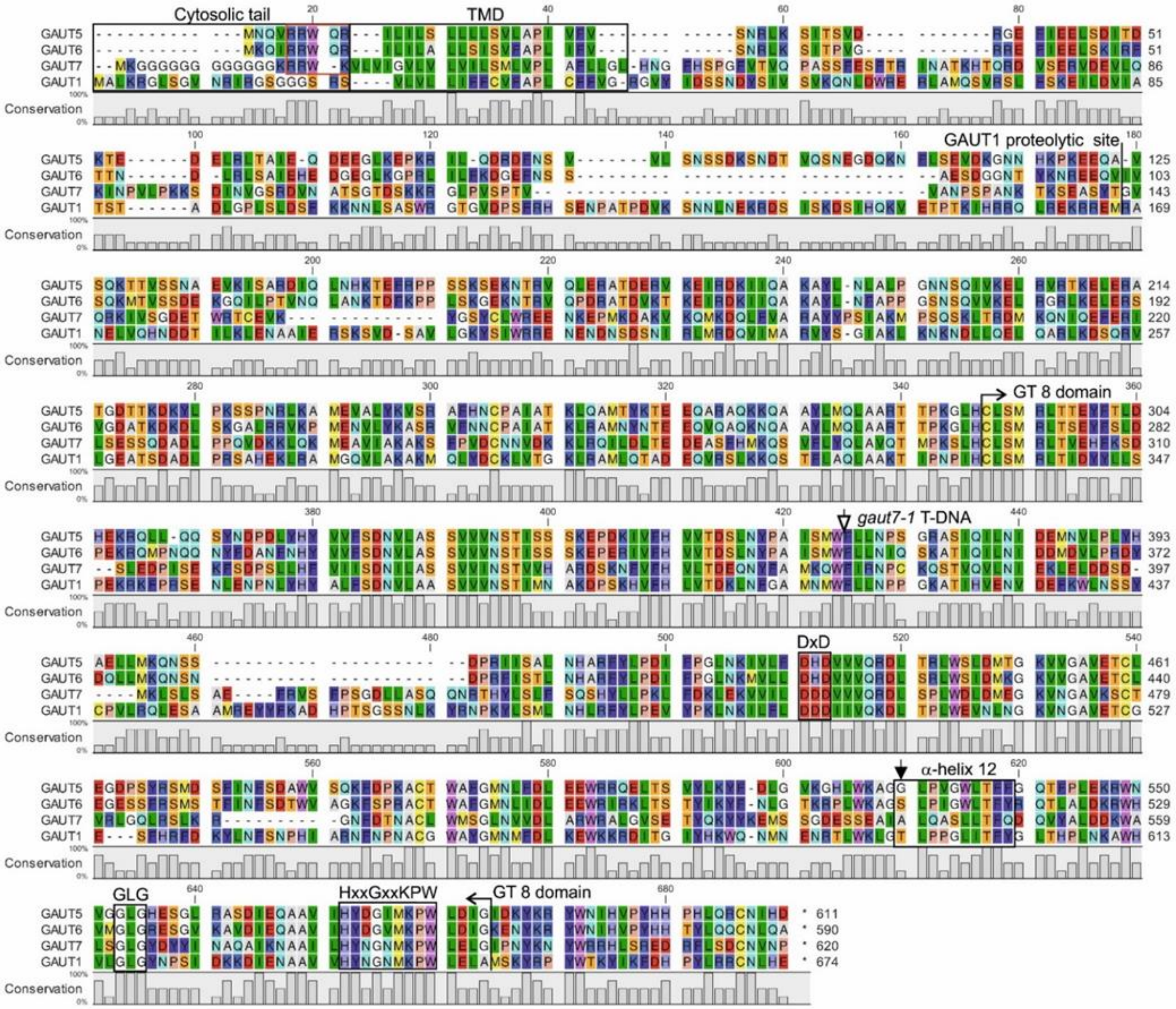

Supplementary Figure 2. Amino acid sequence alignment of GAUT1, GAUT5, GAUT6, and GAUT7, related to Figure 3.
Conserved regions (GT8 domain, DxD motif, GLG motif, HxxGxxKPW motif, α-helix 12) and structural motifs (predicted cytosolic tails and transmembrane domain, TMD) are indicated by arrows and boxes. The predicted nucleophilic residues are marked by a black arrow. The T-DNA insertion site in the *gaut7-1* mutant is marked by an open arrow.

Supplementary Figure 3

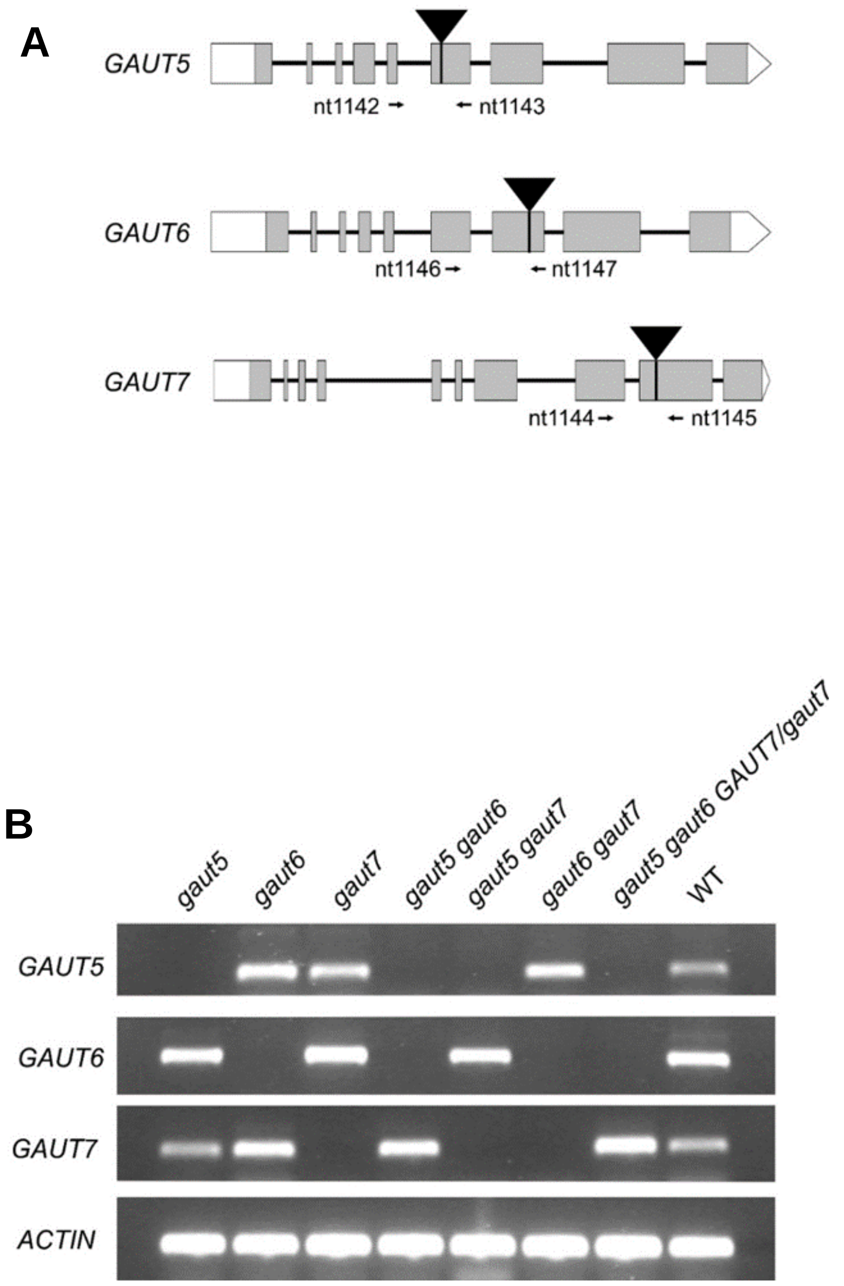

Supplementary Figure 3. RT-PCR analysis of the *gaut* mutants, related to Figure 3
RT-PCR analysis of the *gaut5*, *gaut6*, *gaut7* homozygous single, homozygous double, and *gaut5*$^{-/-}$ *gaut6*$^{-/-}$ *gaut7*$^{+/-}$ triple mutants.
(**A**) Gene models and primer binding sites. Primer sequences are available in Supplementary Table 2.
(**B**) RT-PCR products, generated from RNA isolated from rosette leaves, separated by agarose gel electrophoresis. Total RNA was isolated and equal amounts of RNA were used as the template. RT-PCR of the *ACTIN* transcript is shown as a positive control.

Supplementary Figure 4

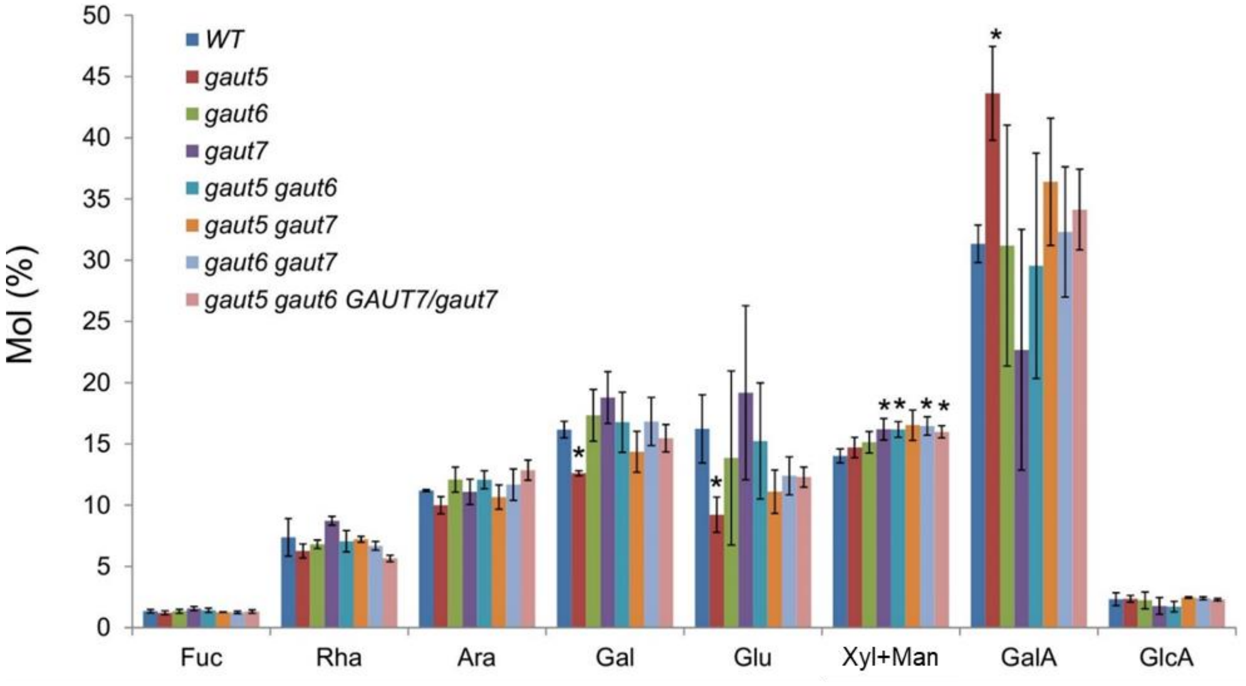

Supplementary Figure 4. Monosaccharide composition analysis of gaut mutants.
Total alcohol insoluble residues (AIR) were extracted from rosette leaves of the *gaut5*, *gaut6*, *gaut7* homozygous single, homozygous double, and triple *gaut5*$^{-/-}$ *gaut6*$^{-/-}$ *gaut7*$^{+/-}$ mutants followed by enzymatic starch removal. The destarched materials were hydrolyzed by trichloroacetic acid and analyzed by HPAEC PAD as described in Stranne et al. (2018). Xyl+Man represents the combined Xyl and Man residues which cannot be distinguished by the method used. Significant differences are shown by * (Student´s t-test, $p<0.05$, $n=4$, error bars represent SEM).

Supplementary Figure 5

A

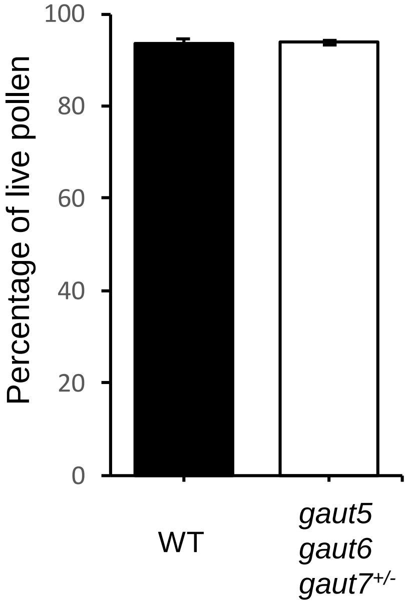

B

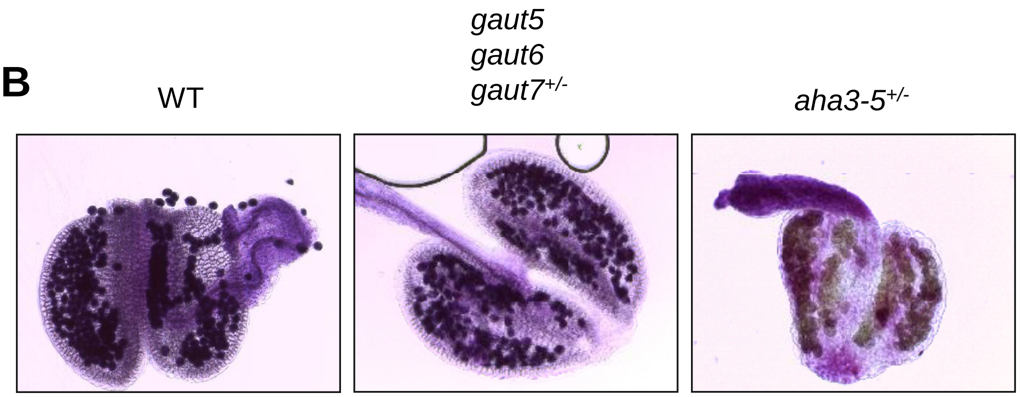

Supplementary Figure 5. Viability of pollen from the WT and *gaut5*$^{-/-}$ *gaut6*$^{-/-}$ *gaut7*$^{+/-}$ mutant (**A**) Pollen harvested from flowers was stained with fluorescein diacetate (n>100). There was no statistically significant difference in the percentage of live pollen in the WT compared to the heterozygous triple mutant (One-way ANOVA, p>0.05).
(**B**) Analysis of anthers using Alexander staining. Both the WT and the heterozygous triple mutant pollen showed purple staining, indicating pollen viability. As a control, staining of anthers from the *aha3-5*$^{+/-}$ plants showed green staining in approximately half of the pollen due to pollen lethality as previously reported (Robertson et al., 2014).

Supplementary Figure 6

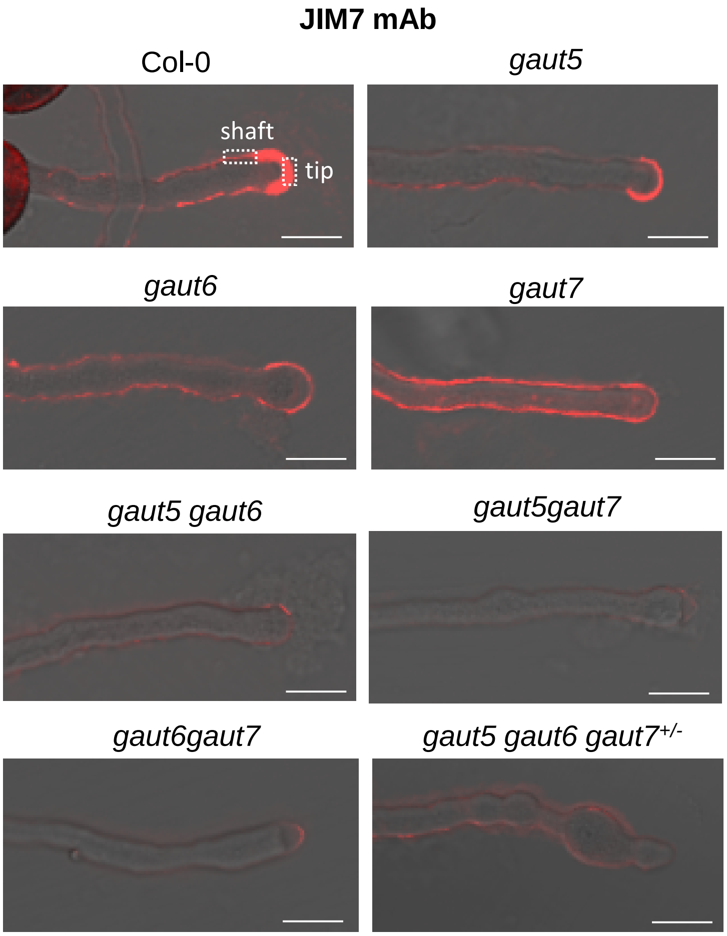

Supplementary Figure 6. Immunolocalization analysis of *gaut* pollen tubes Immunolocalization highly methyl esterified HG in pollen tubes of *gaut* mutant combinations using JIM7 antibody. The dashed square indicates the regions used for quantification in Fig. 5D. At least 8 pollen tubes were analyzed for each genotype in two independent experiments. Overlay with the DIC channel. Scale bars = 10 μm.

Supplementary Figure 7

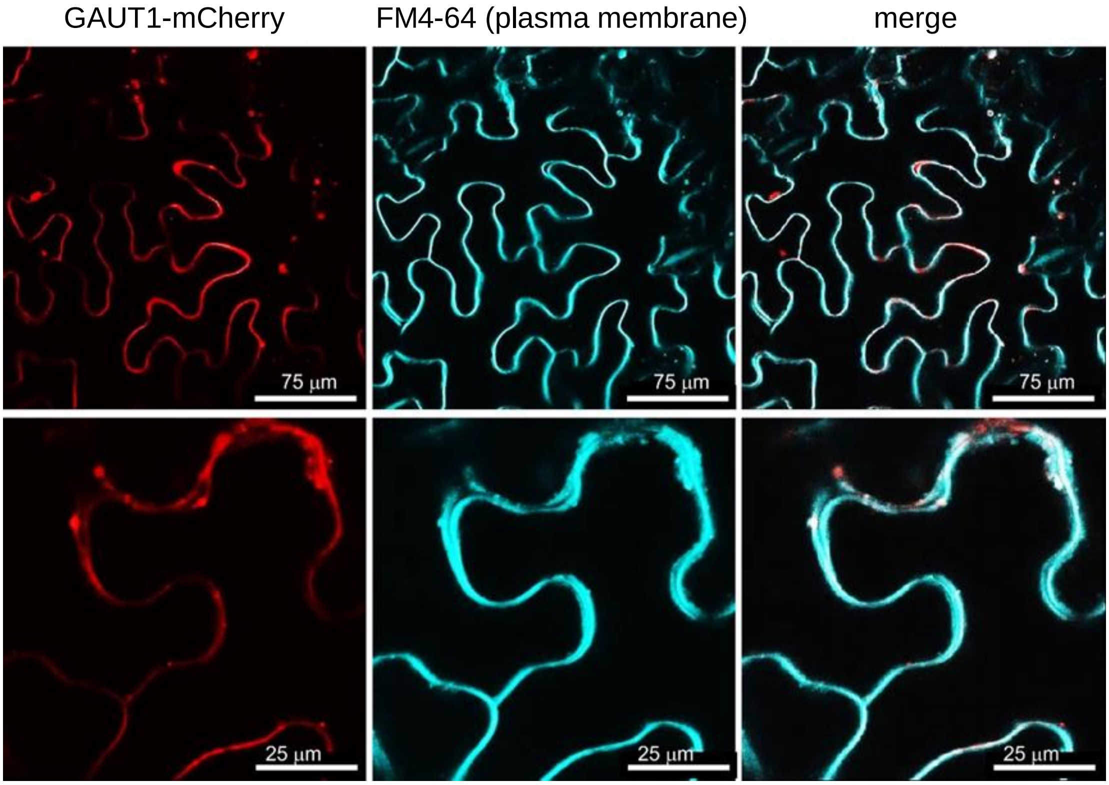

Supplementary Figure 7. Subcellular localization of GAUT1-mCherry, GAUT1-mCherry, a fusion protein wherein GAUT1 was C-terminally fused with mCherry, was transiently expressed in N. benthamiana for 3 days and the subcellular localization was determined by confocal scanning laser microscopy. Left column, mCherry signals. Middle column, FM4-64 for plasma membrane staining. The right column, merges of the mCherry and FM4-64 signals. The top row, imaging using ×1000 magnification. The bottom row, zoom-in images.

Supplementary Figure 8

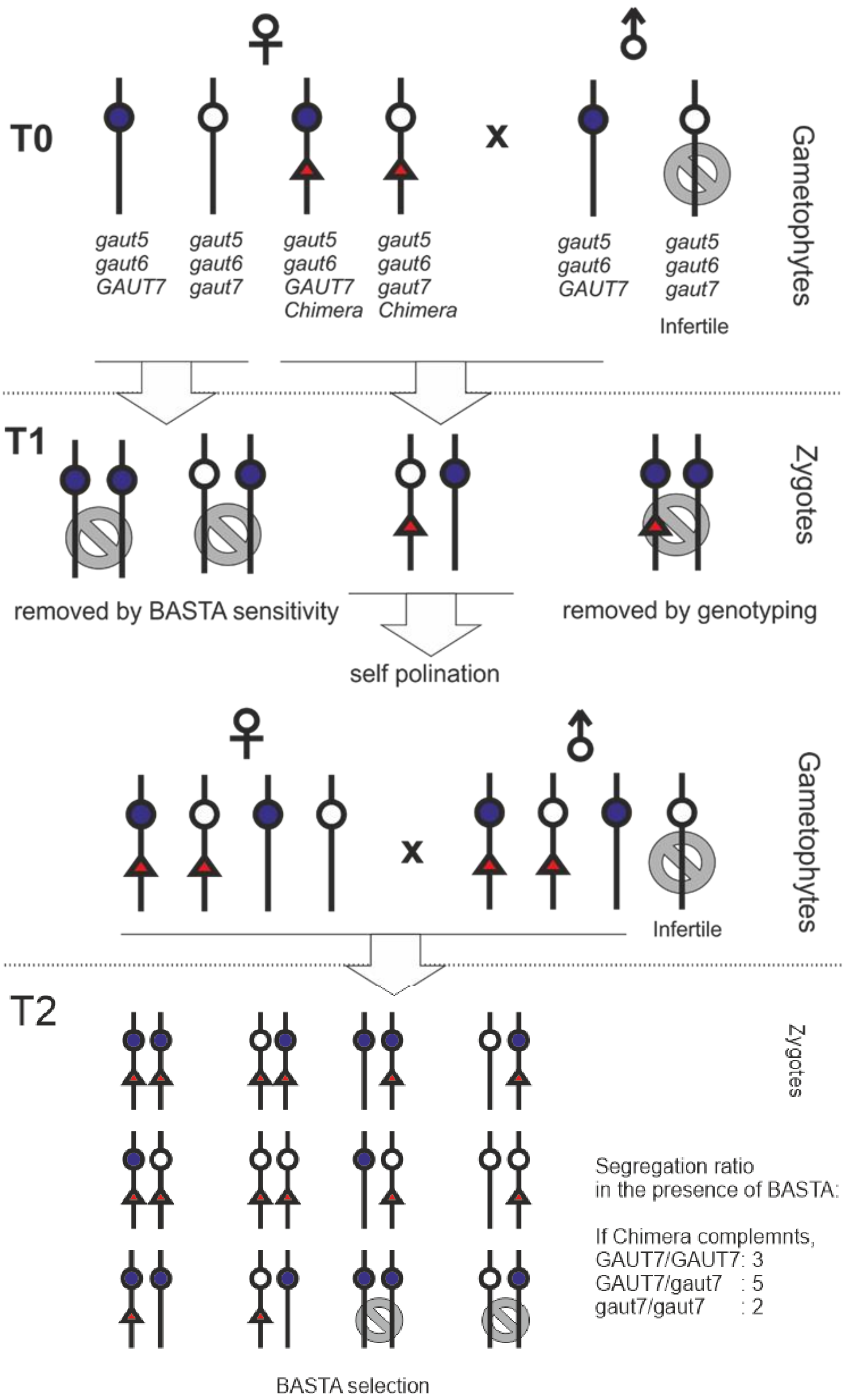

Supplementary Figure 8. Segregation scheme of the *pGAUT1::XYLT:GAUT1* chimera
Schematic illustration of the segregation of the *gaut7* locus in gametophytes of the triple mutant (*gaut5*$^{-/-}$ *gaut6*$^{-/-}$ *gaut7*$^{+/-}$) transformed with the chimera.
The *gaut7*$^{+}$ locus, indicated as *GAUT7*, is shown as a blue-filled circle, the *gaut7*$^{-}$ locus as an open circle, and the *GAUT1* chimera as a red-filled triangle.

Supplementary Table 1

Segregation of herbicide resistance in the T3 population of the 897-10 line, the negative control line, and the WT. * Significant different based on Chi square test ($p < 0.001$)

Transgenic lines (T2 generation)	*n*	Herbicide resistance in the T3 population: Observed values (expected value based on the assumption of a single insertion)	
		Resistant	Sensitive
897-10	156	120 (117)	36 (39)
Negative control	125	96 (94)	29 (22)
WT*	42	0	42

Supplementary Table 2

Primer sequences

Primer	Sequence (5'-3')
**Production of entry vectors by BP recombination**	
attR1-GAUT4 fwd	TCGAAGGAGATAGAACCatgatggtgaagcttcgcaa
attR2-GAUT4 rvs	GTACAAGAAAGCTGGGTCaggattgatgttgcattctc
attR1-GAUT9 fwd	TCGAAGGAGATAGAACCatggcggtggccttccgtgg
attR2-GAUT9 rvs	GTACAAGAAAGCTGGGTCgagaccaaaattgcacatct
**Generating promoter GUS fusions**	
GAUT5 fwd	AAG CTT GAG AAG CGA GCG ATA CCG TAA AGA G
GAUT5 rev	TCT AGA CAA TGT GCG TAA CTT CTC AGA GAA CGC
GAUT6 fwd	TCT AGA CTT TCT CTT TAT CTG TTG GGC TTA ATC ACT CAC
GAUT6 rev	GGA TCC GAA TCT ACA AAT AAA AAA AAA TCC CCA AAT CTA TCT TCC
**Generating GFP and YFP controls for pixel mean intensity analysis**	
GAUT6 CTS fwd	GGCTTAAUATGCGTCGATGGCAGAGGATTTT
GAUT6 CTS rvs	GGTTTAAUccATTCGATACGAAAATAAGCG
GAUT7 CTS fwd	GGCTTAAUATGAAAGTTCTGGTGATTGGAGT
GAUT7 CTS rev	GGTTTAAUccAAGACCGAGTAAGAAAGCAA
**Genotyping primers**	
LBb1.3	ATTTTGCCGATTTCGGAAC
GAUT5 LP	CCTTCAATCTGTTAGGAGAGC
GAUT5 RP WT	AATCACAAACCAAAGGAGGAAC
GAUT5 RP	TTGTTTTGTTGTCATTATCGGGG
GAUT6 LP	ACCAACAGTCAACCAACTTGC
GAUT6 RP	TTCAAACCCGGGAATATATCC
GAUT7 LP	GAGTGTGTTCCTCTACCAGC
GAUT7 RP WT	CTCGCTGGACTACAACGTCA
GAUT7 RP	GGAACTCCGCAGACAAAGAC
**Primers for RT-PCR**	
gaut5 fwd	ACCACAGTAAGCTCGAATGC
gaut5 rvs	CCAGCTCTTTCGTTCGAACT
gaut6 fwd	TGCAACAAAGGACAAGGACT
gaut6 rvs	AGCCAAAACATTGTCAGAGAAGA
gaut7 fwd	GCATCGTCCGTTGTGATCAA
gaut7 rvs	GGAACTCCGCAGACAAAGAC
Actin fwd	ACATTGTGCTCAGTGGTGGA
Actin rvs	TCATACTCGGCCTTGGAGAT
**Cloning into DUALmembrane vectors via the SfiI restriction site**	
GAUT1 fwd SfiI	GGCCATTACGGCCATGGCGCTAAAGCGAGGGC
GAUT1 rvs SfiI	GGCCGAGGCGGCCTTATTCATGAAGGTTGCAACG
GAUT5 fwd SfiI	GGCCATTACGGCCATGAATCAAGTTCGTCG
GAUT5 rvs SfiI	GGCCGAGGCGGCCTCAATCGTGAATGTTGC
GAUT6 fwd SfiI	GGCCATTACGGCCATGAAACAAATTCGTCGATGG
GAUT6 rvs SfiI	GGCCGAGGCGGCCTCAAGCTTGAAGATTGCACTG
GAUT7 fwd SfiI	GGCCATTACGGCCATGAAAGGCGGAGGCGGTGG
GAUT7 rvs SfiI	GGCCGAGGCGGCCTCAAGGATTCACGTTACAG
Anp1 fwd SfiI	ATTAACAAGGCCATTACGGCCATGAAGTATAATAACAGAAAACTC
Anp1 rvs SfiI	AACTGATTGGCCGAGGCGGCCCTAGTTTCTATCAGGGTCGAAGTC
**Generating GAUT1 chimeric proteins**	
GAUT1 CTS fwd	GGCTTAAUATGGCGCTAAAGCGAGGGC
GAUT1 CTS rvs	GTCGCTTUTCCTGCACCTCCCTTAAAAGAATCAAG
GAUT1 C-region fwd	AAGCGACUGGAGGTGCAGGACGGGCAAATGAGTTAG
GAUT1 C-region rvs	GGTTTAAUCCTTCATGAAGGTTGCAACG
GMII CTS fwd	GGCTTAAUATGCCGTTCTCCTCGTATATCG
GMII CTS rvs	GTCGCTTUCCGATTGGATCTGGAGGTAAG
XYLT CTS fwd	GGCTTAAUATGAGTAAACGGAATCCG
XYLT CTS rvs	GTCGCTTUCTTTGTAAATCCATTCCCGA
GAUT1 chimera C-region fwd	AAGCGACUCGGGCAAATGAGTTAGTTC
GAUT1 rvs	GGTTTAAUTTATTCATGAAGGTTGCAACG
proGAUT1 fwd	GGCTTAAUCTTACTGTTGTTTGACCC
proGAUT1 USER rvs	GGTTTAAUGCTGAGGTTTAATTAAGCCTCAGCCCCTTGTACAGCTCGTCCAT
